# Micro magnetic resonance spectroscopy for noninvasive metabolic screening of mammalian embryos and oocytes

**DOI:** 10.1073/pnas.2424459122

**Published:** 2025-07-28

**Authors:** Giulia Sivelli, Arthur Barakat, Kathryn B. Marable, Guillaume Gruet, Serena L. Bitetti, Barry Behr, Valentina Lodde, Alberto Maria Luciano, Carolina Herrera, Michelle Blom, Marco Grisi

**Affiliations:** ^a^Annaida Technologies, École Polytechnique Fédérale de Lausanne Innovation Park C, Lausanne 1015, Switzerland; ^b^Departments of Obstetrics and Gynecology, Stanford University, Stanford, CA 94305; ^c^Reproductive and Developmental Biology Laboratory, Department of Veterinary Medicine and Animal Sciences, University of Milan, Milan 20122, Italy; ^d^Paladis, Zurich, Switzerland; ^e^Animal experimentation unit, Centre hospitalier universitaire vaudois, Lausanne 1011, Switzerland

**Keywords:** micro MRS, metabolic fingerprinting, non-invasive embryo screening, Magnetic Resonance Spectroscopy, single-cell MRS

## Abstract

Analyzing cellular health and metabolism without compromising cell integrity is a major challenge. We present a noninvasive technique using micro magnetic resonance spectroscopy (micro MRS) for nondestructive metabolic fingerprinting at the single-cell scale. This is an application of micro MRS to bovine preimplantation embryos (~8 cells) and oocytes (single cell), with measurements performed on a total of over 150 samples. Among various applications, this method holds significant potential for assisted reproductive technologies (ART), where metabolic assessments of preimplantation embryos could improve treatment outcomes. Early results indicate that classification models using micro MRS data effectively distinguish embryos with high developmental potential and show correlation with oocytes maturity. Furthermore, a multigenerational safety study in a mouse model revealed no adverse effects from embryo exposure to static magnetic field. These findings indicate that micro MRS is a promising, safe tool for assessing embryo metabolism, potentially improving the efficiency and outcomes of ART.

## Significance

Live birth rates in human assisted reproductive technologies (ART) remain suboptimal, despite significant advancements in assessing preimplantation embryo viability and developmental potential. This study presents a groundbreaking, noninvasive approach to in vitro embryo screening using micro magnetic resonance spectroscopy (micro MRS). By capturing a biochemical fingerprint in a nondestructive and biocompatible manner, micro MRS has the potential to revolutionize embryo selection processes, enhance ART success rates, and contribute valuable insights to developmental biology and reproductive medicine.

Since the first reported live birth conceived through in vitro fertilization in 1978, assisted reproductive technologies (ART) have become increasingly utilized worldwide to help couples conceive (1–4). Unfortunately, ~65% embryo transfers fail, making ART treatments disappointingly inefficient (5). Since implantation failure seems to be mainly due to embryonic factors (6), the assessment of the viability and developmental competence of embryos is a critical step in the process (7, 8). The gold standard for preimplantation embryo analysis is through morphological assessment (including time-lapse imaging) and preimplantation genetic testing for aneuploidies (PGT-A) (1). Genetic testing, while informative, is expensive, requires specialized personnel, and involves an invasive biopsy. Both genetic and morphological assessments offer valuable insights but provide only limited information about the embryo’s metabolic state, which could be a key factor in its developmental potential (9–13).

Here, we present micro magnetic resonance spectroscopy (micro MRS) as a noninvasive method for metabolic profiling of preimplantation embryos. With MRS, it is possible to observe endogenous compounds in vivo, representative and uniquely linked to cellular health and metabolism (14). For instance, MRS has emerged as a powerful tool for noninvasive detection of biochemical compounds, including fatty acids (FAs) in the form of mobile lipids (15–23), crucial for cellular health and development (24–28). Fatty acids are recognized as indicators of cellular health and function, participating in many crucial metabolic reactions. They are essential for maintaining energy homeostasis, membrane integrity, and signaling pathways, providing ATP through β-oxidation within mitochondria (25, 29–32). Recent investigations into embryo metabolism have highlighted the importance of energy reserves (essentially stocked in mobile lipids) for embryo survival until successful implantation (24, 26, 27, 33–39).

Recently, we showed that micro MRS can be used to acquire spectra from single preimplantation embryos, with fatty acids (FAs) being the dominant signals in bovine embryos (19). This technique relies on microchip-based MRS probes, which integrate on-chip excitation/detection electronics (40–45) and 3D microprinted structures to ensure precise sample placement (17–19). In this work, we employ a 4-channel device, equipped with temperature control, to perform in vivo parallel micro MRS spectroscopy on four samples simultaneously (see Materials and Methods, and *SI Appendix*, Fig. S1). The increased throughput provided by the quadruple-channel setup enabled statistically meaningful population classification studies. Leveraging this capacity, we recorded 1D ^1^H spectra from over 150 bovine early embryos and oocytes within an effective experimental time of approximately 40 h. The correlation between micro MRS data and the developmental potential of the samples was also evaluated, giving positive indications. Finally, to assess the safety of micro MRS, we conducted a multigenerational mouse study. The results indicated no significant effects on implantation rates, live birth rates (LBR), or the overall health and reproductive potential across three generations.

## Results

Within an hour of averaging time, at a magnetic field strength of 7 Tesla, typical spectra of bovine samples display peaks in regions associated with FAs (*SI Appendix*, Table S1 and Figs. S10–S12), with linewidths averaging 0.12 ± 0.04 ppm, substantial intensity variation between samples (approximately a 13-fold difference), and an average Signal-to-Noise Ratio (SNR) of 22 ± 8 (calculated as peak maximum in the saturated hydrogen region divided by noise SD). A temperature control system is used to minimize any impact on the samples, maintaining the four samples at 37.7 ± 0.5 °C (*SI Appendix*, Fig. S2). Prior to measurements, the magnetic field is shimmed using only the culture medium, and once adjusted, it remains unchanged throughout the day (*SI Appendix*, Fig. S3). Under these conditions, we investigated correlations between MRS profiles and either embryo developmental outcomes or oocyte maturation.

### Classification of Developmental Potential Using MRS Biomarkers in Day 2 Embryos.

**Fig. 1.**
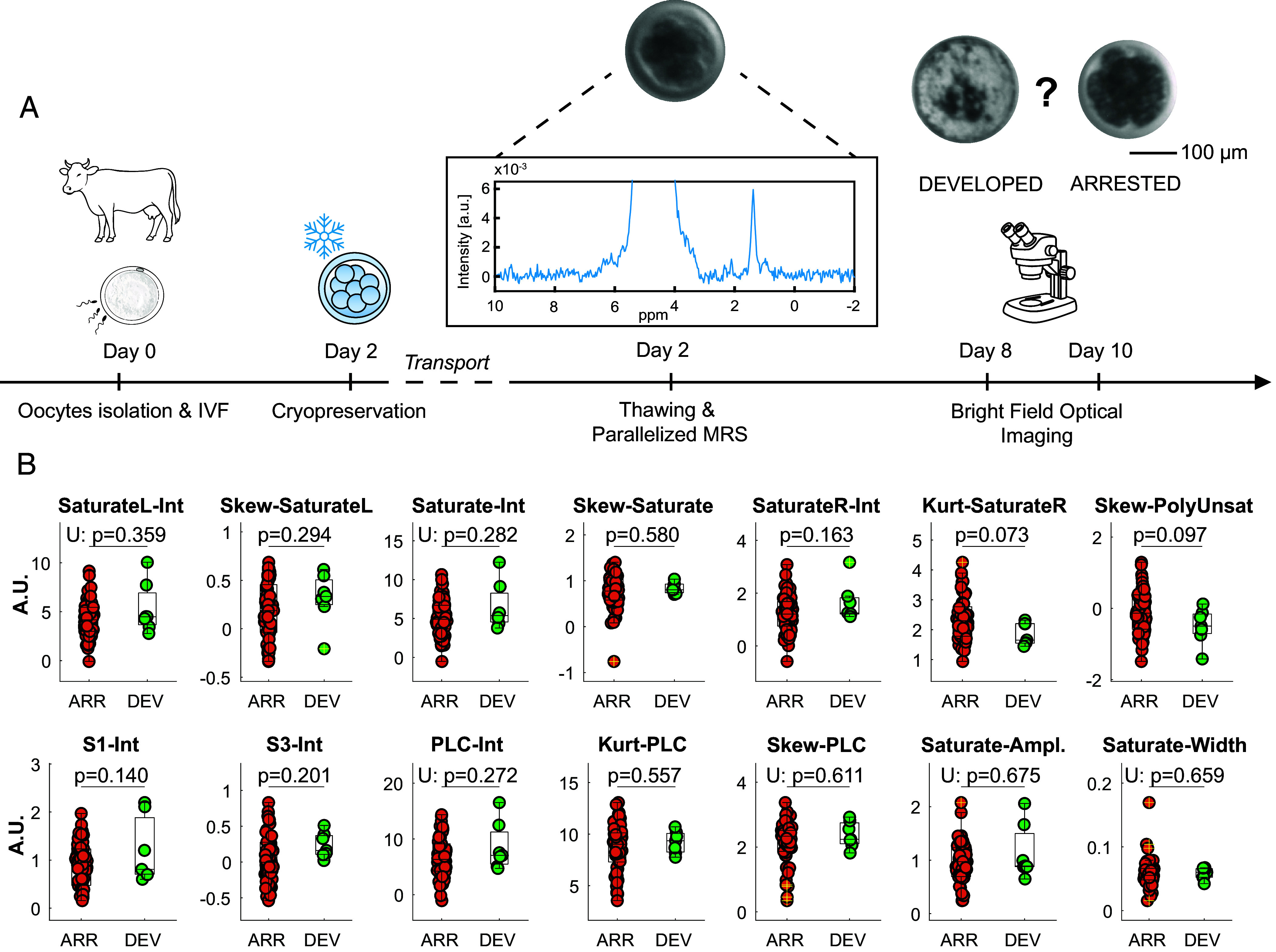
Correlation of MR spectra with developmental outcome in Day 2 (~8-cell) embryos. (*A*) Procedure overview: collection of oocytes and sperm, embryo production via IVF (Day 0), cryopreservation at the 8-cell stage (Day 2), thawing and MRS measurement, and subsequent incubation. Development is assessed on Day 8 and Day 10 by bright field microscopy to categorize embryos as expanded blastocysts (Developed, DEV) or developmentally arrested (Arrested, ARR). (Scale bar, 100 μm.) (*B*) Biomarker quantification for ARR (n = 54) and DEV (n = 7) groups. Normality was assessed with the Lilliefors test, with significance determined by the *t* test or Mann–Whitney U test (U). Biomarkers include intensity (-Int), skewness (-Skew), and kurtosis (-Kurt) within specific chemical shift regions of the spectrum: PLC [0.83 to 2.8], Saturate [1.1 to 1.5], S1 [0.83 to 1.03], S3 [2.23 to 2.36], SaturateL [1.3 to 1.5], and SaturateR [1.1 to 1.3]. Saturate-Ampl and Saturate-Width are derived from Lorentzian fit parameters in the Saturate region. See *SI Appendix*, Tables S1 and S2 for additional information on biomarkers definition.

**Fig. 2.**
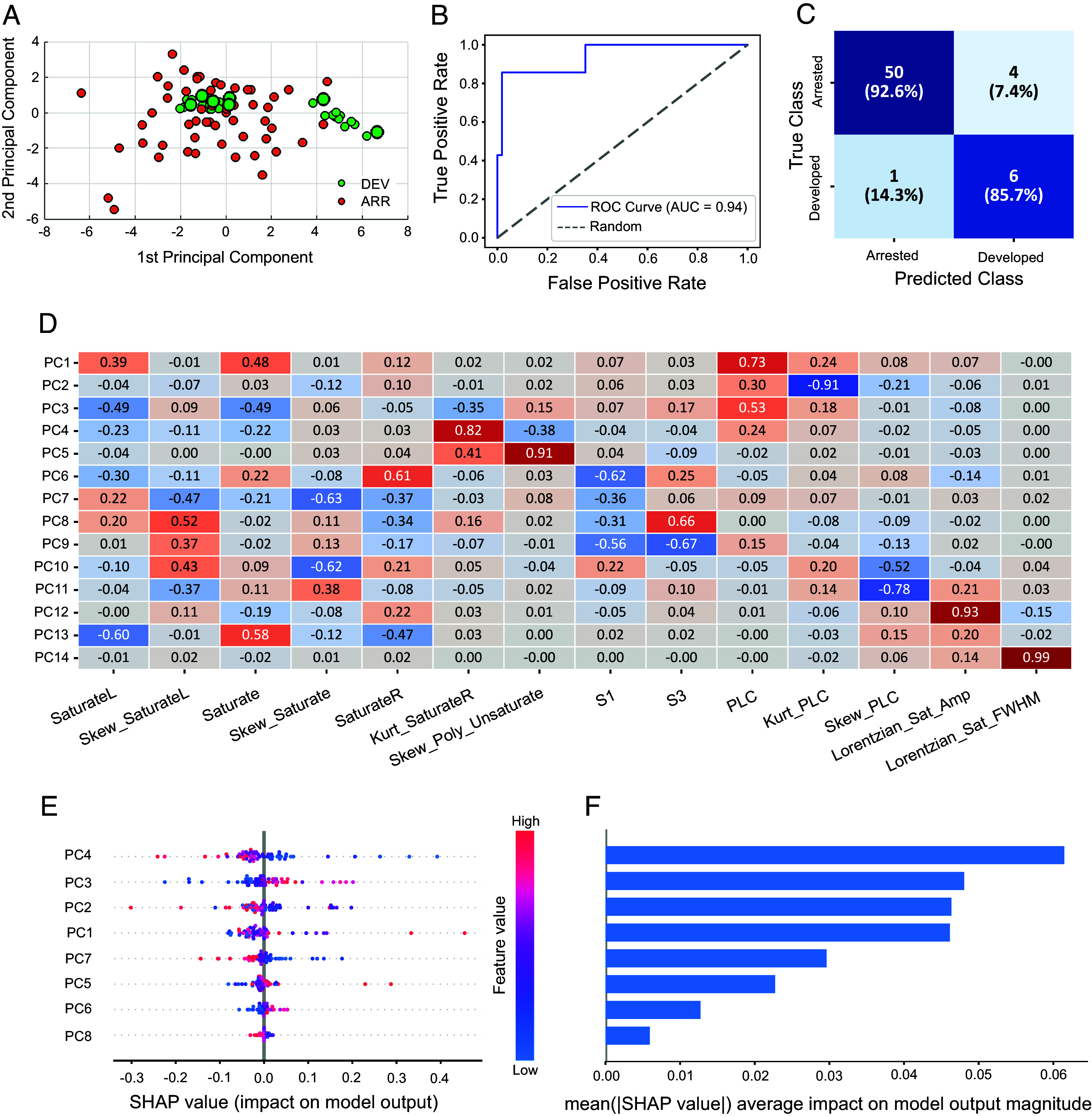
Classification of developmental potential based on 8-cell embryos MRS biomarkers. (*A*) Typical first and second principal components [A.U] observed after applying SMOTE (Synthetic Minority Over-sampling Technique) on the whole dataset to balance it (N_TOT_ = 61, N_ARR_ = 54, N_DEV_ = 7). Red: arrested embryos; big green: original developed embryos; small green: synthetic developed embryos. (*B*) ROC curve with AUC comparison between a dummy classifier (predicting the most frequent class) and our SVM model, both evaluated using a CVLOO strategy. (*C*) Confusion matrix aggregated from the CVLOO predictions of the SVM model. SMOTE was applied exclusively to the training data in each fold to address class imbalance, ensuring no data leakage into the validation set. (*D*) PCA loadings showing the contribution of each original feature to the principal components. PCA was performed on the training data prior to SMOTE application. (*E* and *F*) SHAP summary and bar plots illustrating the first 8 most influential principal components in the model and their effect on prediction outcomes.

Fig. 1*A* shows the experimental procedure where micro MRS is employed to investigate the developmental potential of Day 2 bovine embryos. First, oocytes and sperm are collected, and then, embryos are produced through in vitro fertilization (IVF, Day 0), grown up to Day 2, cryopreserved, and cryoshipped to our laboratory. After that, embryos are thawed, measured on the same day with parallelized single-embryo MRS, and placed in the incubator to continue their culture. At Day 8 and Day 10 of their development (i.e., 6 and 8 d after the MRS measurements), embryos are imaged with a bright field stereomicroscope to determine whether they developed until becoming expanded blastocysts (Developed, DEV group) or arrested their development before reaching this essential milestone (Arrested, ARR group). Fig. 1*B* shows a comparison between developed (DEV) and arrested (ARR) embryos for 14 micro MRS biomarkers computed from 1D ^1^H spectra as intensity, skewness, kurtosis, or amplitude and linewidth of Lorentzian fits using data from predefined chemical shifts regions (*SI Appendix* and *SI Appendix*, Tables S1 and S2). The so-defined biomarkers were validated by an independent experiment where 6 embryos were measured by permuting the physical on-chip channel (*SI Appendix*, Fig. S4), demonstrating they carry sample-dependent information distinct from noise. To further investigate the biomarkers’ value, it was verified that they are independent of the physical channel (*SI Appendix*, Fig. S5) and sensor (*SI Appendix*, Fig. S6) in which the samples are measured. This poses the basis for using these parameters to construct an MRS-based biochemical fingerprint of each embryo. Fig. 2*A* shows a typical scatter plot of the first and second principal components obtained on the whole dataset (N_TOT_ = 61, N_ARR_ = 54, N_DEV_ = 7), where a cluster is visible for the developed embryos (green), supporting the feasibility of training a classifier based on these markers.

To evaluate the potential of MRS biomarkers in assessing embryo developmental competence (ARR vs. DEV), we trained a support vector machine (SVM) classifier. As shown in Fig. 2*B*, the Receiver Operating Characteristic (ROC) curve highlights a clear performance difference between the SVM model and a baseline dummy classifier (the dotted line) that consistently predicts the majority class, resulting in a random guess. The SVM achieved a high area under the curve (AUC = 0.942), outperforming the dummy model (AUC = 0.5). The SVM model demonstrated robust classification performance across multiple metrics: accuracy = 0.92, balanced accuracy = 0.856, sensitivity = 0.857, precision = 0.60, negative predictive value = 0.98, and F1-score = 0.71 (Fig. 2*C*). Fig. 2*D* displays the PCA loadings, illustrating the weight of each original feature in the derived principal components. Model interpretability was further enhanced through Shapley additive explanation (SHAP) analysis (Fig. 2 *E* and *F*). The SHAP summary and bar plots, showing the 8 most important PCs, indicate that the fourth principal component (PC4) is the most influential in driving the model’s predictions. Specifically, lower PC4 values, corresponding to low kurtosis of the right side of the saturate peak, are associated with a higher likelihood of embryo development, whereas average to high PC4 values tend to shift toward embryo undergoing an arrested state. Despite the modest sample size and the limitations related to sample cryopreservation (*Discussion*), these findings suggest that MRS biomarkers possess predictive value about the developmental competence of embryos.

### Micro Magnetic Resonance Analysis of Oocyte in vitro Maturation.

**Fig. 3.**
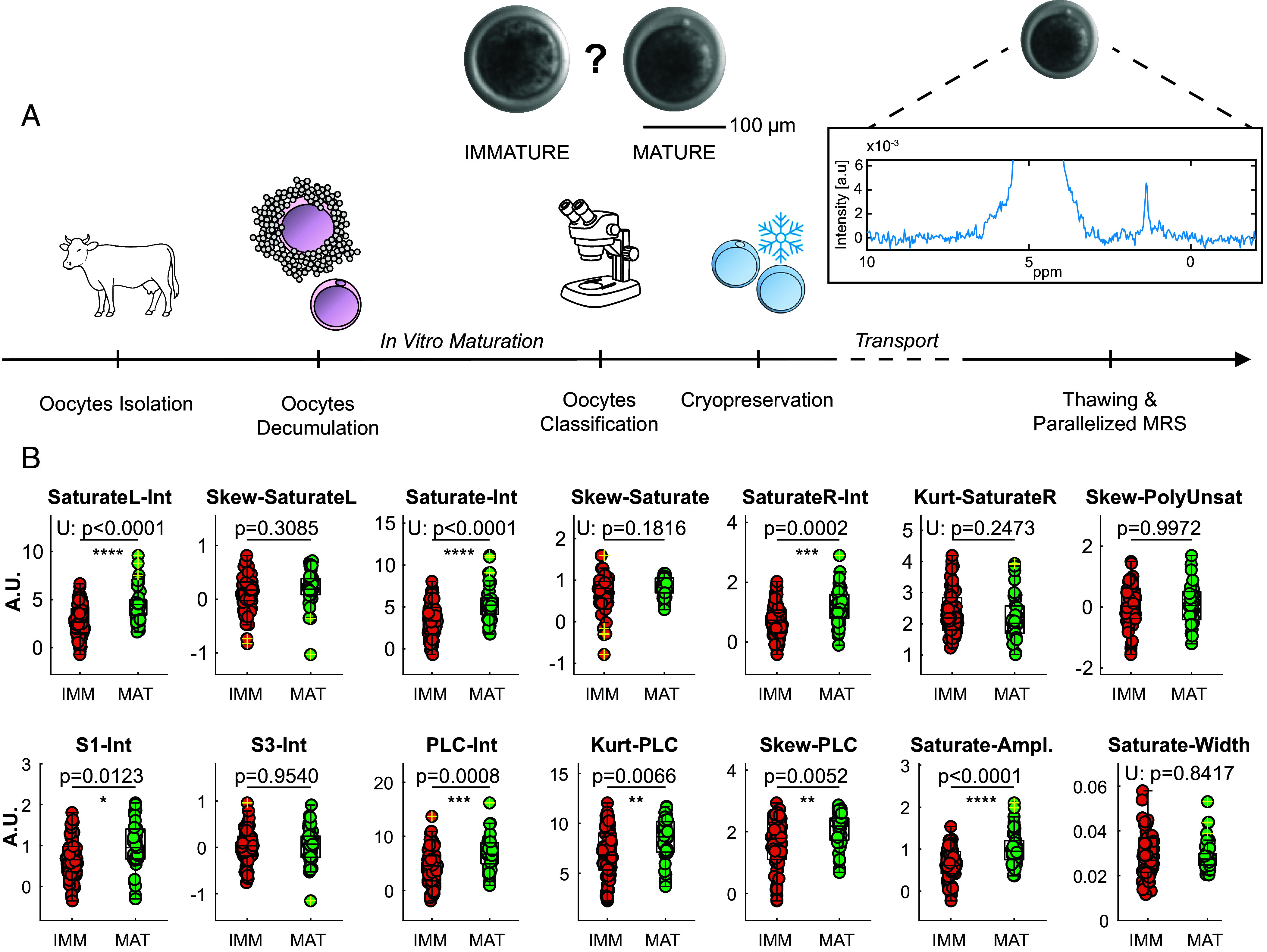
Magnetic Resonance analysis of oocytes maturation. (*A*) Procedure overview: ovaries are aspirated, oocytes denuded of cumulus cells and incubated for In Vitro Maturation (IVM). Maturation is assessed by the presence of a polar body under a bright field microscope, categorizing oocytes as immature (IMM) or mature (MAT). The oocytes are cryo-preserved post-assessment, transported to our laboratory, thawed, and measured with parallelized single-oocyte Magnetic Resonance Spectroscopy (MRS). (Scale bar, 100 μm.) (*B*) Biomarkers quantification for immature (IMM, n = 63) and mature (MAT, n = 39) oocytes. Normality assessed with Lilliefors test, with significance determined by unpaired *t* test or Mann-Whitney U Test (U). Biomarkers include Intensity (-Int), Skewness (-Skew), and Kurtosis (-Kurt) within specific chemical shift regions of the spectrum: PLC [0.83 to 2.8], Saturate [1.1 to 1.5], S1 [0.83 to 1.03], S3 [2.23 to 2.36], SaturateL [1.3 to 1.5], SaturateR [1.1 to 1.3]. Saturate-Ampl and Saturate-Width are derived from Lorentzian fit parameters in the Saturate region.Saturate-Ampl and Saturate-Width are derived from Lorentzian fit parameters in the Saturate region. See *SI Appendix*, Tables S1 and S2 for additional information on biomarkers definition.

In this second experiment, micro MRS spectroscopy is applied to individual cow oocytes. Fig. 3*A* shows the experimental procedure: Oocytes are harvested from ovarian tissues and denuded of cumulus cells prior to incubation for in vitro maturation (IVM). After a maturation period of 22 h, the oocytes are evaluated with a bright field microscope to determine whether they displayed a polar body, an indicator of full maturation. Two cohorts were obtained: immature (IMM, n = 63) and mature (MAT, n = 39). The samples are then cryopreserved and transported to our laboratory. Once thawed, they are measured on the same day with parallelized micro MRS. Fig. 3*B* shows the analysis of MRS biomarkers for both immature (IMM) and mature (MAT) oocytes, in analogy to the biomarkers used in the Day 2 embryo investigation (Fig. 1*B*). In this second experiment, several biomarkers showed statistical significance, with p-values indicating a clear difference in the average values between the two cohorts. For instance, mature oocytes exhibit significantly higher intensity in all biomarkers tied to Saturated protons (“SaturateL-Int” and "Saturate-Int,” as well as “Saturate-Ampl.”, *P* < 0.0001). Other significant differences include a reduction in skewness for the whole region with lipid signals (“Skew-PLC”, *P* = 0.0052) and increased intensity for “S1-Int” and “PLC-Int” (*P* = 0.0123 and *P* = 0.0008, respectively). Additionally, “Kurt-PLC” shows increased kurtosis in the lipid’s signals (*P* = 0.0008), indicating sharper spectral profiles in mature oocytes. Extending to machine learning as previously described, we conducted an analysis equivalent to the one used for Day 2 embryos to determine whether it was possible to train a classifier on this different dataset and samples. *SI Appendix*, Fig. S9*A* shows a typical scatter plot of the first and second principal components obtained on the whole dataset (N_TOT_ = 102, N_IMM_ = 63, N_MAT_ = 39), where a cluster is visible for the mature oocytes (green). *SI Appendix*, Fig. S9*B* presents the ROC curve, highlighting a performance difference between the SVM model and a baseline dummy model. The SVM achieved a moderate area under the curve (AUC = 0.687), outperforming the dummy model (AUC = 0.5), and moderate classifying performance across multiple metrics: accuracy = 0.69, balanced accuracy = 0.56, sensitivity = 0.52, precision = 0.60, negative predictive value = 0.72, and F1-score = 0.56 (*SI Appendix*, Fig. S9*C*). *SI Appendix*, Fig. S9 *D*–*F* illustrates how individual features contributed to the model’s characterization.

### Safety Assessment of Magnetic Field Exposure in Two-Cell Mouse Embryos.

**Fig. 4.**
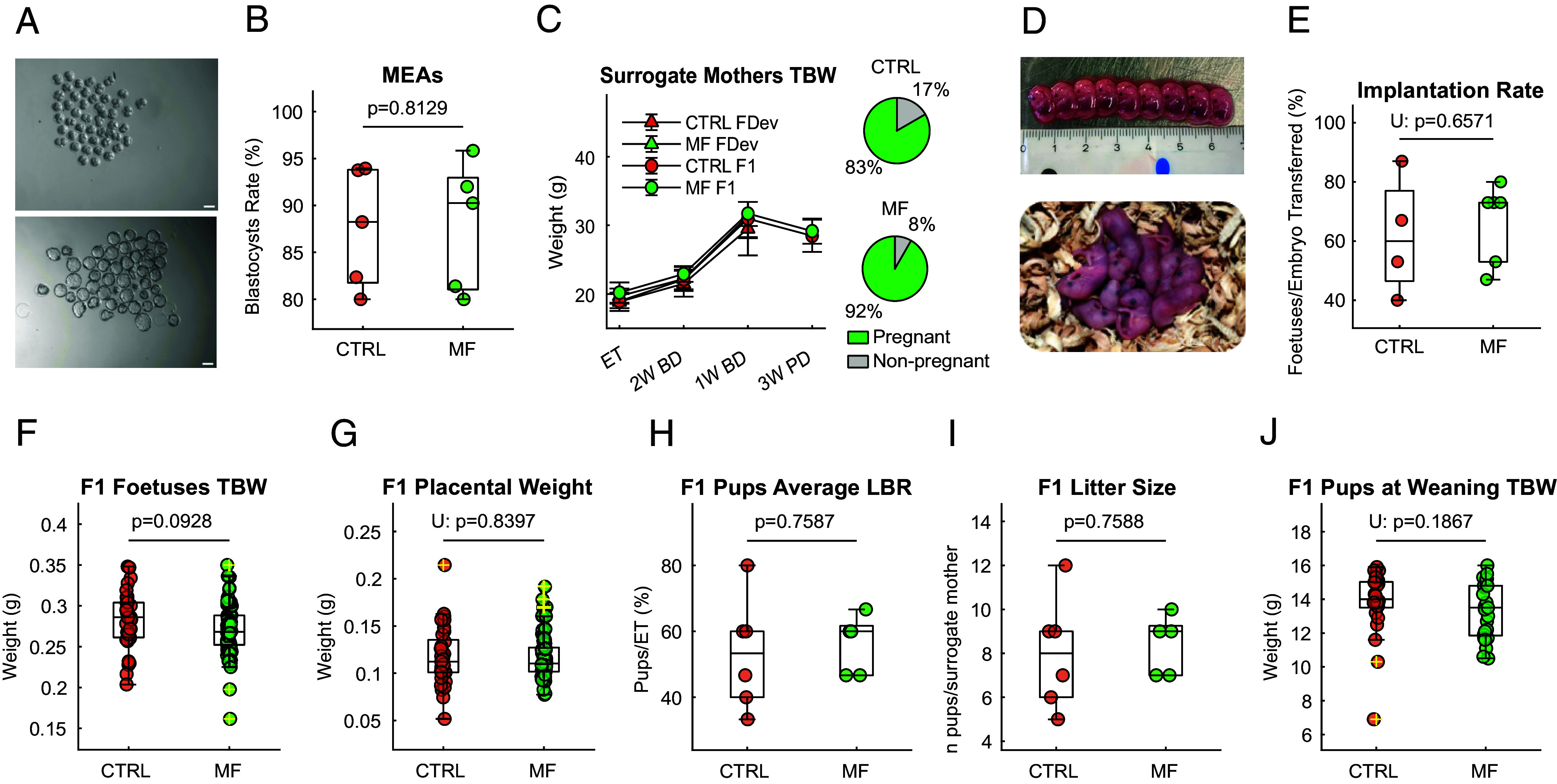
Main IVF outcomes of Static Magnetic Field (MF) exposure on 2-cell embryos. Bars represent SD with statistical significance set at *P* < 0.05, indicated on top of each comparison. Normality was assessed with the Kolmogorov-Smirnov test, and significance was determined by an unpaired *t* test or Mann-Whitney U Test (indicated with U:). (*A*) Representative images of 2-cell embryos exposed to Static MF developing to blastocyst. (*B*) Mouse Embryo Assay (MEA) comparing blastocyst rates, showing no significant difference (*P* = 0.1930) between embryos exposed to a 9.4 Tesla static MF versus CTRL; n = 187 CTRL, n = 227 MF embryos, across 5 experiments. (*C*) *Left*: body weight progression of surrogate mothers at embryo transfer, during pregnancy, and 3 wk post-delivery. F0-Fdev = mothers killed before delivery to assess implantation (n = 12); F0-F1 = mothers kept alive until delivery to assess LBR (n = 12). *Right*: pregnancy rates of surrogate mothers (n = 24, 12 MF, 12 CTRL) as a percentage post-embryo transfer. (*D*) *Top*: representative image of ED14 fetuses from the Static MF exposure group. Bottom: Representative image of F1 pups from the Static MF exposure group. (*E*) Implantation rate as the percentage of fetuses at embryonic day (ED) 14 against the number of transferred embryos (n = 15 embryos transferred per surrogate mother). No significant difference observed (*P* = 0.6571, n = 10 surrogate mothers, 4 CTRL, 6 MF; n = 37 fetuses in CTRL, n = 59 fetuses in MF). (*F*) Average foetal TBW at ED14 (n = 96, 37 CTRL, 59 MF). No significant difference observed (*P* = 0.0928). (*G*) Average placental weight at ED14 (n = 95, 37 CTRL, 58 MF). No significant difference observed (*P* = 0.8397). (*H*) F1 pups’ average LBR as the percentage of live F1 pups relative to the number of transferred embryos (n = 15 embryos transferred per surrogate mother) and (*I*) Number of F1 pups at delivery from surrogate mothers post-embryo transfer (ET). No significant difference observed (*P* = 0.7587, n = 11 surrogate mothers, 6 CTRL, 5 MF; n = 48 CTRL F1 pups, n = 42 MF F1 pups). (*J*) Average TBW of F1 pups at delivery from surrogate mothers post-ET. No significant difference observed (*P* = 0.1867, n = 11 surrogate mothers, 6 CTRL, 5 MF).

Mouse embryo assays (MEAs) are a standard test to assess toxicity toward embryos (46). The MEA involves exposing mouse embryos to a test substance and assessing their developmental progress, typically requiring over 80% expanded blastocyst formation rates (EBFRs) for a product to be deemed nontoxic. Research has highlighted the sensitivity of MEAs in detecting embryotoxicity in a range of products, from culture media and mineral oils to more novel substances like nanoparticles (47). In Fig. 4 *A* and *B*, we demonstrate no significant difference (*P* > 0.05) in the blastocyst rate percentage (>80%) between control or treated embryos obtained after exposing 2-cell embryos to the static magnetic field in a 9.4 T 400 MHz magnet. After this initial in vitro evaluation, we progressed to an in vivo study to address the potential adverse effects of static MF exposure on preimplantation embryos. This dataset was necessary to understand whether the transfer of static MF-exposed embryos and the consecutive pregnancy could impact i) the overall health status of the surrogate mothers, ii) the reproductive potential of the mice, and iii) the short- and long-term physiological development of the pups. Various IVF outcomes, alongside more general physiological end points, were recorded for both mothers and their pups to evaluate the risks associated with embryo exposure to the static magnetic fields required by MRS. In Fig. 4 *C*–*J*, we report the primary effects in the F1 generation, with the findings for F2 and F3 in the supplementary figures in panel *SI Appendix*, Fig. S8. Overall, the primary IVF outcomes measured post–embryo transfer of Control (CTRL), or embryos exposed to the Static Magnetic Field (MF) were comparable across groups. Fig. 4*C* shows how the surrogate mothers’ total body weight (TBW) increased through pregnancy for both cohorts after embryo transfer, a sign of successful embryo transfer and pregnancy establishment. Furthermore, the pregnancy rates remained comparable between the two groups and, in both cases, were above 80% (Fig. 4 *C* *Right*). No significant difference (*P* > 0.05) was found when assessing implantation rates (Fig. 4*E*), LBRs (Fig. 4*H*), and the number of F1 pups at delivery (Fig. 4*I*). Complementary parameters were also assessed, such as the TBW of the ED14 fetuses (Fig. 4*F*) and their placenta (Fig. 4*G*), as well as the weight of the F1 pups at weaning (Fig. 4*J*). Five main organs from F1 pups and their surrogate mothers (*SI Appendix*, Fig. S7 *A* and *B*) were evaluated for histopathological changes and weighed (*SI Appendix*, Fig. S8 *K* and *L*). The pregnancy rates of the F1 and F2 mothers expressed as percentages of pregnant mothers after natural mating (NM) were comparable between CTRL and MF groups (*SI Appendix*, Fig. S8 *C* and *F*).

## Discussion

This study reports high-throughput MRS data from single oocytes and preimplantation embryos at the cleavage stage, demonstrating the applicability of MRS to classification based on metabolic fingerprinting. More specifically, we explored the application of micro MRS as a technique for assessing mammalian embryos, offering direct insights into their biochemistry in a nondestructive way. Our findings indicate that its application to preimplantation embryos shows potential for evaluating developmental competence without impacting the embryo’s development, pregnancy rates, and the offspring’s health.

We recognize some limitations in the current work. First, the analysis of Day 2 bovine embryos was conducted after cryopreservation, a procedure that is known to significantly reduce the survivability of early-stage embryos in the bovine model. This reduction in survivability is particularly pronounced at this stage of preimplantation development, as it coincides with genome activation, a critical process that may be adversely affected by cryopreservation (48). This limitation introduces a confounding variable that may partially affect outcomes. Second, the MRS measurements were conducted over a 1 h period with temperature regulation, but without gas control. However, we verified that the experimental procedure has a minimal impact when the developmental ability is compared to a parallel control culture (*SI Appendix*, Table S3). These limitations may influence the statistical representation of embryo development observed in our Day 2 experiment. Still, of the 61 embryos analyzed with micro MRS, those that survived and expanded over the subsequent 6 to 8 d arguably represent a highly viable subgroup, allowing for clearer distinctions within the population. In the oocyte study, the potential effects of these limitations are expected to be marginal, as their maturation stage was evaluated prior to micro MRS.

Our findings suggest that micro MRS can assess embryo developmental competence and can predict embryo development over several days. This outcome is closely linked to the nature of the lipids detected through micro MRS spectroscopy. The method will “bias” more mobile lipids, i.e., those characterized by a certain degree of molecular tumbling (49), as the tumbling improves line shape and, thus, detectability. This condition is more prominent in intracellular lipid droplets (LDs), which have diameters larger than a few hundred nanometers allowing liquid-like tumbling in their interiors (50). In models of tumors and cell cultures, LDs are recognized markers of apoptosis, necrosis, metabolic diseases, and proliferation capabilities (16, 49, 51–54). LDs are crucial dynamic organelles for oocyte maturation and early embryonic development (24, 26, 39, 55, 56). They are not only energy reservoirs but are also involved in plasma membrane biosynthesis and gene expression modulation (55, 57). Suppression of LDs poses severe impairment to embryonic early development (58), while excessive accumulation is linked to mitochondrial function alteration (59). A recent study linked LD accumulation and mobilization to implantation and postimplantation embryo development (60). These findings indicate that a correct balance of mobile lipids is pivotal for successful oocyte and embryo viability, supporting our data.

Currently, the two most widely utilized methods for assessing embryo quality in ART are morphological evaluation, including microscopy and image analysis, and PGT-A. Morphological evaluation relies on visual assessment, while PGT-A screens for chromosomal abnormalities by the trophectodermal DNA after an embryo biopsy. Although advancements in optical technologies, such as morpho-kinetic algorithms and time-lapse microscopy, have been made, these methods have not been proven to improve embryo transfer success (61, 62). PGT-A provides a snapshot of the embryonic chromosomal status but requires an invasive biopsy procedure that is costly, time-consuming, and involves highly trained personnel to minimize manipulation risks for the embryos (63–67).

An alternative analysis based on spent medium has been proposed. However, noninvasive genetic testing (niPGT) and metabolic analysis through spent media have shown low accuracy rates, making these techniques unsuitable for routine use (68–78). This is due to severe fundamental limitations that apply to spent medium analysis. Crucially, spent medium analysis fails to assess intracellular biomarkers, providing only indirect evaluations. One can only infer biochemical information indirectly through measurements of compound uptake and expulsion by the embryo. Therefore, many relevant endogenous markers (such as LDs or intracellular DNA) are inaccessible through this approach. Additionally, the embryo’s volume (~nL) is over 10,000 times smaller than that of the culture medium (~20 μL), making accurate quantification challenging as useful information is present in very low concentrations against a chemically rich background.

More recently, metabolic minimally invasive assessment of embryos and oocytes like fluorescence lifetime imaging microscopy (FLIM) (79, 80) and hyperspectral imaging (81) have been proposed. Both methodologies have the advantage of targeting endogenous compounds, but they are limited to autofluorescent molecules (82–84). Consequently, the informative value on metabolism is restricted to specific processes, as only subcomponents linked to fatty acids can be observed. A recent clinical trial found no correlation between FLIM and positive embryo development (85). Our study introduces micro MRS as a noninvasive approach to measure small molecules inside embryos, offering direct insights into embryo biochemistry through the detection of hydrogens and their abundance in various chemical configurations. Unlike FLIM and hyperspectral imaging, which are limited to detecting NADH and FAD autofluorescence, micro MRS measures a broader range of molecules.

Our results demonstrate that micro MRS can successfully perform spectroscopy on individual bovine embryos and oocytes at various developmental stages, suggesting its potential for noninvasive metabolic screening. By directly assessing the biochemical composition of embryos through the detection of hydrogen atoms and their distribution in various chemical configurations, this technique provides valuable insights into cellular health and metabolic status. While micro MRS provides valuable metabolic information, its safety has not previously been tested for preimplantation embryos in a mammalian model. Using the mouse embryo assay (MEA) and an extensive in vivo study in the mouse model involving ET, we assessed the potential adverse effects of the exposure. Our findings confirm safety on several end points: We observe no significant impact on implantation rates, LBRs, or mice’s overall health and reproductive potential over three generations. With no adverse effect on embryo development or subsequent generations, our data suggest the procedure’s safety for preimplantation embryos. These findings are aligned with previous studies showing that the static magnetic field alone has no or minimal effects on cell growth and genetic toxicity, regardless of magnetic field density (86, 87). Additionally, we estimate that less than 1 mJ of radiofrequency energy at 300 MHz is exchanged with the embryo over 1 h of measurement. The specific absorption rate is estimated to be below 100 mW/kg, well within the safety limits required for in vivo MRI in clinical examinations (88, 89).

In summary, micro MRS is a promising, noninvasive method for assessing embryo quality by measuring metabolic biomarkers directly within the embryo in a nondestructive and biocompatible manner. Our safety validations and machine learning analyses demonstrate the feasibility of implementing this metabolic embryo assessment to support fertility care by guiding the selection of the best embryos for transfer. We discussed the advantages of MRS over traditional methods, which often require invasive procedures and/or provide limited information. Further improvements in sample control, additional safety validations, and clinical trials are needed to fully assess the relevance of MRS in empowering ART.

## Materials and Methods

### Ethical Approval.

All procedures involving live mice were carried out in accordance with Swiss animal welfare legislation and approved by the local Institutional Animal Care and Research Advisory Committee under license VD3671–33532.

### Animals and Husbandry.

All procedures described in this study performed in vivo in the mouse model occurred at the Animal Facility of the Centre Hospitalier Universitaire Vaudois (CHUV), Lausanne, Switzerland. The mice were maintained in individually ventilated caging systems (floor area 440 cm2, GM500, Tecniplast) under controlled standardized conditions: temperature 22 ± 2 °C, relative humidity 50 ± 5%, and artificial light (12 h light, 12 h dark; light on at 6:00 a.m.). Sterilized commercial softwood granulate bedding was used (SAFE, SAFE select). The mice received a commercial pellet diet (SAFE, SAFE 150) and autoclaved water ad libitum. The microbiological status of the mice colony was examined as recommended by Federation of Laboratory Animal Science Associations (FELASA), and the mice were free of the listed microorganisms. All embryos were purchased from Janvier Labs (Le Genest-Saint-Isle, France) as cryopreserved at 2-cell stage and were imported to our lab based at EPFL (École Polytechnique Fédérale de Lausanne) Lausanne, Switzerland. Live mice were constantly monitored by the CHUV animal facility personnel and body weight was recorded at preestablished timepoints as described in the results section. The animal facility personnel checked daily the behavior of the animal, the fur, and the skin to detect any possible sign of disease. This monitoring allowed to assess the general condition of the animals (emaciated, thin, adequate/good condition, obese) and to record any external lesions (skin lesions, fur loss or discoloration, and subcutaneous masses). Mice were killed by intraperitoneal injection of at least 200 mg/kg sodium pentobarbital solution. Mice were immediately processed as described below for necropsy, total body weighing, and single organ weighing.

### Mouse Preimplantation Embryo Preparation and Culture.

Cryopreserved 2-cell B6D2F1 preimplantation mouse embryos were purchased from Janvier Labs, (Le Genest-Saint-Isle, France) and stored in our cryogenic biobank. On the day of the experiment, the embryos were thawed according to the manufacturer’s protocol (Janvier Labs, Le Genest-Saint-Isle, France). Morphological integrity was evaluated under a bright field stereomicroscope and morphologically intact embryos with two distinct blastomeres and an intact zona pellucida were used for the experiments. When embryos were intended to use for in vitro mouse embryo assays, they were transferred to standard culture conditions in 50 µl drops of pre-equilibrated M16 culture medium (Sigma-Aldrich, REF M7292) overlayed with mineral oil (Sigma-Aldrich, REF M5310) at 37 °C, with 5% CO2 under 90% humidity until they reached the blastocyst stage. When embryos were used for in vivo embryo transfer, thawing occurred 1 h prior to the static magnetic field (MF) exposure. Embryos were loaded in a 5 mm glass tube adapted for placement in the bore of a 9.4 Tesla Magnet and loaded with 1 mL of pre-equilibrated culture medium at 37 °C. Static MF exposure was performed for 1 h with temperature control previously set at 37 °C. A control group of embryos followed the same procedure but was not exposed to the magnetic field.

### Mouse Embryo Transfer.

Recipient females B6CBAF1/J are bought between 6 and 7 wk of age. Vasectomized NMRI males are bought at 8 wk and used until they are 1 y old depending on their breeding record. To induce pseudopregnancy, nulliparus recipient females were mated to vasectomized males with proven sterility. The next morning, plug-positive females were considered as day 0.5 pseudopregnant and used as surrogate dams. Embryo transfer was performed under general anesthesia achieved by intraperitoneal injection of anesthetic solution (Xylasol (20 mg/mL) /Ketasol (50 mg/mL): mix 2 ml Ketasol + 0,8 ml Xylasol -> up to 11 mL with water) 0.1 mL per 10 g of body weight. The eyeballs were covered with ophthalmic gel (Lacryvisc) to protect the cornea. As soon as the toe pinch reflex had disappeared, embryos were collected for the transfer in a capillary making three air bubbles with a mouth pipet in a minimal amount of medium. The lower part of the back of the mouse was humidified with a few drops of ethanol 70% and the back wiped with Betadine solution. A small incision less than 1 cm in the skin with fine dissection scissors was made, 1 cm away from the spinal cord and at the level of the last rib. The skin was slided to the left or the right until seeing the fat pad through the body wall to make a small incision. The ovary was pulled out until the oviduct and the upper part of the uterus attached to the fat pad was clearly visible. The mouse was placed under the stereomicroscope. A clamp clipped onto the fat pad and lay it down over the middle of the back so that the ovary and oviduct remain outside the body wall. A hole was made in the bursa (membrane surrounding the ovary and oviduct) with two forceps. An edge of the infundibulum was picked up and the transfer pipet containing the embryos was inserted into the natural opening for the transfer. The clamp was unclipped, and the uterus, ovary, and oviduct were repositioned within the body cavity. Embryo transfer was performed with 15 embryos unilaterally into the oviduct. The skin was sutured with a clip, and 0.5 ml of NaCl 0.9% was injected intraperitoneally. Betadine cream was applied on the wound. Postsurgery, recipients were allowed to recover on a warming pad at 37 °C, were monitored until they regained consciousness and locomotor abilities, and were kept singly until the birth of the progeny, after which they remained with their pups until weaning. The numbers of litters and pups born were recorded. For analgesic support, recipients received paracetamol during 3 to 4 d in water bottle (250 mg/125 mL of water).

Two sets of cages were created according to the timing of mouse termination:

#### Set-1: Fetal development cohort (FDev).

Mice were killed 1 wk before delivery (at embryonic day 14 of development) to assess the implantation rate of the embryos transferred into recipient mothers. Upon necropsy, we assessed the number of fetuses present in the uterine cavity. Following necropsy, all the fetuses and organs sampled from the surrogate mothers were weighed fresh immediately after dissection and then processed for postmortem histopathological analysis.

#### Set-2: First-generation cohort (F1).

Surrogate mothers and part of their pups were killed 3 wk postdelivery to assess the LBR and no adverse effect on the first generation of mice (F1). Part of the F1 pups were kept alive for the breeding of F2. The remaining F1 pups and surrogate mothers were killed at weaning to perform postmortem histopathological analysis as for Set 1. All the organs dissected were weighed fresh immediately after dissection. Part of the F2 pups were kept alive for breeding of F3. The remaining F2 pups and respective mothers were killed at weaning and all the organs dissected were weighed fresh immediately after dissection. All the F3 pups and respective mothers were killed at weaning and the TBW of each mouse was annotated. In addition to postmortem analysis, we also collected data on live mice such as TBW during pregnancy for the mothers, average litter size and LBR. The surrogate mothers that failed to achieve pregnancy after embryo transfer were included in the analysis for the assessment of pregnancy rates and efficiency of the ET procedure but were then excluded from the rest of the study. Necropsy, specimen collection weighing, and organ processing for histology. The list of organs examined with the main purpose of histopathology evaluation of tissue samples is very much inspired from necropsy performed in toxicological studies as described in the ICH S5(R3) guidelines. The major steps are as follows: examination of the live animal, killing of the animal, weighing of the entire animal, opening of the abdominal cavity and dissection of liver and spleen, opening of the thoracic cavity and dissection of heart and lungs, opening of the skull, and dissection of the entire brain. Weighing of the fresh organs was performed immediately after the dissection. Organs were placed in prelabeled 50 mL tubes (Nunc™ 50 mL Conical Sterile Polypropylene Centrifuge Tubes, Thermo Fisher Scientific REF 339653) immersed in the fixative (10% Neutral buffered PFA, Sigma-Aldrich REF HT501128) as quickly as possible after sampling, macroscopic examination, and weighing. Following the recommendations from the Society of Toxicologic Pathology (STP), we sampled the liver, spleen, heart, brain, and lungs as representative organs. In all cases, organs were weighed free of surrounding fat and connective tissues. It is important to remove these tissues in a standardized manner and without inducing any damage or artifact to the tissue. 24 h post**–**collection and fixation, organs were taken to the histopathology lab of SV (Sciences de la vie) department at EPFL where they were processed for trimming, paraffin infiltration, paraffin embedding, sectioning, H&E (hematoxylin and eosin) staining, and glass slide mounting. Finally, the tissue sections were submitted for histopathological evaluation by the accredited personnel collaborating with EPFL.

### Micro MRS Setup and Experimental Details.

Our micro MRS device, illustrated in *SI Appendix*, Fig. S1, features a 7 Tesla spectrometer modified with a microchip-based probe and a 3D-printed system for sensor positioning (*SI Appendix*, Fig. S1*A*). The sensors, coated with parylene-C for liquid contact and biocompatibility (90), are prepared under a microscope and inserted into the magnet for analysis (Movie S1). *SI Appendix*, Fig. S1*B* shows the sensor’s microsystem, housed in a plastic chamber holding 1.5 mL of culture medium and designed to host and simultaneously analyze up to 4 samples. Samples are secured on the sensing regions (defined by on-chip microcoils) using a 3D-printed structure with holding cups, facilitating precise placement and retrieval via a standard micropipette. *SI Appendix*, Fig. S1*C* displays the microcoil sensing map. The microchip, a custom CMOS circuit fabricated with TSMC 0.18-micron technology, integrates the necessary electronics for magnetic resonance as previously published (44, 45). *SI Appendix*, Fig. S1*D* presents the electronics schematics, featuring a power amplifier and heterodyne receiver connected to the coils via a switch. During analysis, excitation pulses are synchronized with a decoder for coil selection, allowing sequential measurements within a single experiment. The free-induction decay signals are collected from each coil to obtain 1D ^1^H spectra of each sample (*SI Appendix*, Fig. S1*E*). Acquisition was performed on NI device. The experimental parameters were setup as 520 ms repetition time, 100 ms acquisition per coil, 131,072 points, and 9,600 averages for approximately 80 min of experimental time. Parameters have been determined in pilot experiments where the signal-to-noise ratio on a few samples was maximized. Water suppression was not applied, as it may introduce artifacts that could compromise small-signal analysis and, in our nanoscale setup, acquisition sensitivity (not dynamic range) limits SNR, making water suppression unnecessary. Additionally, the water peak provides a stable internal reference without affecting SNR.

### Sample Handling for MRS Measurements.

Fig. 1*A* shows the experimental procedure where micro MRS is correlated to the developmental potential of D2 bovine embryos. MRS chamber was loaded with 2 mL of pre-equilibrated BO-IVC (IVF-Bioscience, UK) medium and checked for possible air bubble presence under a stereomicroscope. When present, air bubbles were removed by gently pipetting. Samples were removed from the culture dish and placed in each microwell (4 in total) present in the sensor using a Cook Medical tip and pipette (Movie S1). The MRS chamber was then closed, and the sensor connected to a manual slide & lock mechanism to position the device into the MRS magnet. MRS measurements were performed for a total time of 80 min. A picture of each embryo before and after MRS measurement was taken using a Drawell stereomicroscope with 1X objective to further confirm correct embryo positioning inside the microwells on the chip.

### Bovine Sample Production and Culture.

Bovine sample production was kindly performed by collaborators in Zurich, Switzerland. In vitro embryo production (IVP) and cryopreservation were carried out at Paladis (Zurich, Switzerland), while oocyte production and cryopreservation were performed at the University of Zurich (UZH, Zurich, Switzerland). Cryopreservation of both oocytes and preimplantation embryos was performed by vitrification protocol using the KITAZATO vitrification kit. Thawing of embryos or oocytes was conducted 3 h prior to MRS measurement using the KITAZATO warming kit.

For early-stage embryo production, bovine ovaries were obtained from a local abattoir and transported to the laboratory within 2 h in sterile saline maintained at 26 °C. All media used for embryo IVP were sourced from IVF-Bioscience, UK. Follicles were aspirated using a sterile needle, and oocytes with cumulus cells were isolated. These oocytes underwent in vitro maturation (IVM) at 38.5 °C under 5% CO2 and 6% O2 in humidified air for 24 h. Following IVM, the oocytes were fertilized with cryopreserved bull semen in fertilization medium. Postfertilization, the oocytes were washed, decumulated, and incubated in BO-IVC medium at 38.5 °C under 5% CO2 and 6% O2 for embryo development. Embryo development was monitored using a stereomicroscope until Day 2 postfertilization. On Day 2, embryos were cryopreserved using the vitrification method and shipped to our laboratory for analysis.

For oocyte production, the same procedures were followed up to the isolation of oocytes with cumulus cells. All oocytes were denuded prior to IVM to simulate the oocyte manipulation procedures commonly used in clinical settings for human oocyte isolation. After a maturation period of 22 h, the oocytes were evaluated with a bright field microscope to determine whether they displayed a polar body, an indicator of full maturation. Three distinct groups of oocytes were provided: M2 (matured oocytes postdenudation with 22 h of IVM, indicated as MAT in Figs. 3 and 4), NMPC (denuded oocytes that failed to mature in vitro, as assessed by the absence of the first polar body, 22 h postdenudation), and DEG (completely degenerated oocytes that failed to mature in vitro and were left in culture for 48 h postdenudation). NMPC and DEG are pooled as immature oocytes (IMM). Oocytes were cryopreserved using the vitrification method and shipped to our laboratory for analysis.

Immediately after warming, the samples were transferred to pre-equilibrated BO-IVC medium at 38.5 °C under 5% CO2 and 6% O2 in humidified air for 3 h before MRS measurement. For the embryos, after warming, they were randomly assigned to either the control or MRS group. Following MRS analysis, the embryos were cultured for an additional 10 d in individual drops of 50 µL pre-equilibrated BO-IVC medium overlaid with BO-OIL in a humidified incubator at 38.5 °C with 5% CO2 and 6% O2.

For the oocytes, MRS was performed on all samples, which were then discarded immediately after the measurements.

### SVM-Based Classification Pipeline.

To assess the efficacy of MRS biomarkers in classifying embryos and oocytes, we employed a SVM classifier, chosen for its simplicity and interpretability, particularly advantageous given the limited size of our dataset. Due to constraints in data availability and to ensure a robust estimation of model performance in the absence of an independent test set, we utilized leave-one-out cross-validation (LOOCV). In LOOCV, each validation fold consisted of a single data point, with all remaining data used for training, with a number of fold equal to the dataset size. To address class imbalance and optimize variance explained by each feature, we applied a machine learning pipeline within each fold: First, the training set was standardized, then balanced using the synthetic minority oversampling technique (SMOTE), and subsequently subjected to dimensionality reduction through principal component analysis (PCA). PCA and standardization parameters derived from the training set were applied to the validation set within each fold, ensuring consistent data transformations and preventing contamination from synthetic data in validation predictions. Model performance was evaluated using a comprehensive set of metrics for our analysis: accuracy, balanced accuracy (to account for class imbalance), area under the ROC curve (AUC), precision (reflecting the reliability of positive predictions), sensitivity (identification of true positive embryos), negative predictive value (NPV, reliability of negative predictions), and F1-score (harmonic mean of precision and sensitivity). To further interpret the predictive behavior and quantify the importance of individual features, we employed SHAP. Hyperparameters, steps to minimize overfitting, and further information concerning the model are detailed in the *SI Appendix*. All code, datasets, and additional plots on the dataset are available at the following link: (https://doi.org/10.5281/zenodo.15187904) (91).

### Statistical Analysis.

All results are presented as mean ± SD where n represents the number of samples used for each experiment. Statistical analysis was performed using GraphPad Software V9.0 and/or Matlab. Statistical significance was set at *P* < 0.05. Normality was tested with the Kolmogorov–Smirnov or Lilliefors test. For normally distributed data, parametric tests were used. Specifically, when a single variable was compared between two groups, a two-tailed unpaired t-test was applied. For non-normally distributed data, either an unpaired t-test with Welch’s correction or a Mann–Whitney U test was used.

## Supplementary Material

Appendix 01 (PDF)

Movie S1. Sensor preparation protocol.

## Data Availability

Source Codes data have been deposited in GitHub; Zenodo (https://doi.org/10.5281/zenodo.15187904) (91). Some other data available (Spectroscopy Raw Data have a dimension of a few hundreds MB. These are provided upon request.).
