# Supplementary material for: Micro magnetic resonance spectroscopy for noninvasive metabolic screening of mammalian embryos and oocytes
Source: Proc Natl Acad Sci U S A. 2025 Jul 28;122(31):e2424459122. doi: 10.1073/pnas.2424459122 (PMC12337278; doi:10.1073/pnas.2424459122)
Supplement: Supplementary file 1 — Appendix 01 (PDF) [file pnas.2424459122.sapp.pdf]

Supporting Text

All code, datasets, and water linewidth plots are available at the following link: (https://doi.org/10.5281/zenodo.15187904)

Supplementary materials

Signal preprocessing

During acquisition, sets of 800 time-domain signals were averaged in real time, and this averaging procedure was repeated 12 times per biological sample, yielding 12 final averaged spectra per sample. Time-domain filtering was then applied to the averaged signals to reduce noise and suppress acquisition-related artifacts. The filtered time-domain data were subsequently transformed into the frequency domain using Fourier transformation. The frequency axis was calibrated based on the position of the residual water peak, ensuring alignment across samples. Automatic phasing was performed to correct phase distortions, followed by linear phase correction for finer adjustment. Spectra were then aligned and averaged across replicates to further improve the signal-to-noise ratio. A linear baseline correction was applied to remove low-frequency drifts and offsets. Finally, spectra were normalized by scaling the water peak intensity to unity.

Biomarker description (Tables 1-2)

To extract relevant information from our spectral data, we defined and computed biomarkers representing specific spectral signals corresponding to distinct lipid chemical profiles detailed in Table 1. These biomarkers encompass defined spectral ranges associated with the saturated lipid peak, unsaturated lipid peak, two smaller peaks of potential biological interest (S1 and S3), and the proportional lipid content (PLC). For each of these defined ranges, we calculated three descriptive metrics: the integral of the spectral signal, skewness, and kurtosis. The integral represents the total sum of the spectral intensity within a specified range. Skewness measures the asymmetry of the spectral intensity distribution, indicating whether the distribution is biased toward lower or higher intensity values. Kurtosis describes the sharpness or flatness of the spectral distribution, indicating whether the spectral peak is more pointed, or flatter compared to a normal distribution. Additionally, we performed a Lorentzian curve fitting specifically for the saturated lipid peak, extracting both its amplitude and linewidth parameters. A summary of all biomarkers and their computed metrics is presented clearly in Table 2.

Model's hyperparameters and overfitting prevention

When working with scarce data, model overfitting can be particularly problematic, as insufficient data limits the ability to rigorously demonstrate the absence of overfitting. To mitigate this risk, we implemented several precautionary steps. First, we selected a single, interpretable classifier—Support Vector Machine (SVM)—for both classification tasks. The simplicity and limited hyperparameter set of SVM minimize the potential for overfitting. We deliberately chose hyperparameters based on domain-specific requirements and analytical objectives. For the initial model predicting cow embryo states, we set hyperparameters to C = 10, kernel = radial basis

function (RBF), and classification threshold = 0.15. The high value of C was chosen to ensure the inclusion of all features, the RBF kernel facilitated flexible adaptation to feature clusters, and the low threshold addressed class imbalance. Although applying SMOTE on our training dataset partially mitigates the class imbalance issue, we intentionally set a low threshold to ensure prioritization of minority classes. With minimal exploratory adjustments, this careful selection of hyperparameters contributes to reducing overfitting risks. Under ideal conditions with ample data, hyperparameter optimization would be systematically performed, and results would be validated using a robust independent test set. To further validate our model's stability, we performed an additional hyperparameter optimization using *hyperopt*, an established optimization library. *hyperopt* identified slightly different hyperparameters (C = 7, kernel = RBF, threshold = 0.25), yielding excellent performance metrics with only a single misclassification per class: accuracy = 0.97, balanced accuracy = 0.86, sensitivity = 0.88, precision = 0.87, negative predictive value (NPV) = 0.98, and F1-score = 0.88. These results strongly suggest that our initial model had not overfit.

We applied the same analytical pipeline to our second predictive model, selecting similar hyperparameters (C = 10, kernel = RBF, threshold = 0.5). Due to the minor imbalance in the dataset for this second task and the application of SMOTE, adjusting the classification threshold was deemed unnecessary. Applying *hyperopt* optimization to the second model identified alternative hyperparameters (C = 7, kernel = RBF, threshold = 0.6), resulting in a slight improvement in performance: accuracy = 0.70, balanced accuracy = 0.55, sensitivity = 0.44, precision = 0.65, NPV = 0.71, and F1-score = 0.52. These consistent but modest improvements further confirm that our modeling strategy effectively minimized the risk of overfitting.

Temperature Control Calibration (S2)

Temperature was controlled using a customized heating plate underneath the sensor designed by Okolab Srl. The plate temperature is set and controlled through a feedback loop controller (H401-T-Penny). To ensure that the sample region would be kept close to 37.7 °C (the ideal temperature for bovine embryos), a calibration was made by using a thermocouple fixed on top of the microchip sensing region. By this methodology, the equilibrium temperature is repeatable within ± 0.5 °C around the target 37.7 °C, with a plate-to-microchip offset of about 1.4 °C (i.e., plate temperature set to 38.8 °C to ensure sample temperature stays below target temperature). Additionally, the sensor takes < 16 minutes to recover from cooling during transfer from the microscope to the MRS system, and its equilibrium root mean square (RMS) over the experimental time is below 0.06 °C. By experimental protocol, the heater plate was set to 38.8 °C at the beginning of each measurement day and allowed to equilibrate for > 20 minutes before measurements began.

Shimming Procedure (S3)

Before each day of measurements or after any change of sensors, the culture medium chamber is filled, and the NMR signal measured with no sample in place. If the linewidth of the water signal was greater than 17Hz (0.06 ppm) for any coil, the X, Y, Z, and Z2 shim coil currents were adjusted until all linewidths were within this limit. Figure S2 shows an example of a shimming setup prior

measurements. An acceptance criterion of ~17 Hz (~0.06 ppm) is consistent with prior nanoliter-scale *in vivo* experiments using single-channel devices, where optimal per-sample shimming yielded ~0.25–0.4 ppm in microorganism eggs [15] and human microtissues [16]. In the present multi-channel setup, the linewidths achieved on embryos and oocytes approach the best reported values *in vivo* at this scale (~0.12 ppm), indicating that residual broadening is dominated by sample heterogeneity, not hardware limitations. Reproducibility across channels and sample orientations further confirms system robustness (see S4-S5).

Chemical shifts and tentative assignment

Table S1 indicates chemical shift assignments of different molecular groups previously found in embryos. By comparison with literature, the link to the embryo fat content and in particular its fatty acids composition can be inferred (among which saturated, mono- and poly-unsaturated protons).

Biomarkers Reproducibility (S4)

To characterize reproducibility, we performed a dedicated experiment involving six 8-cell embryos, repeatedly measured in each of the four channels available in the sensor. Figure S3 shows 14 resulting biomarkers where a sufficient signal-to-noise is obtained to distinguish the embryos from an empty channel and/or among themselves. These same biomarkers are those we defined to construct a fingerprint of bovine 8-cell embryos and oocytes, as well as to train machine learning classifiers.

Channel Dependence (S5)

Biomarkers measured in the 61 8-cell embryos from Fig. 2 are plotted against the ID of the channel in which they have been measured. Figure S4 shows that no evident channel dependence is present in the data.

Sensor Dependence (S6)

During the experiment displayed in Fig. 2 it was necessary to change physical sensor, due to a wrong experimental manipulation of the electronic system. As a result, the dataset has been obtained from two different (but identically designed) 4-channel MRS-sensors.

Biomarkers measured in the 61 8-cell embryos from Fig. 2 are here plotted against the ID of the sensor in which they have been measured. Figure S5 shows that no evident sensor dependence is present in the data.

Pathology Report Summary (S7)

The organs were collected fresh on the day of necropsy. Following dissection and tissue processing, each sample was blindly assessed by a certified pathologist as reported below. Overall liver congestion, spleen EMH, lung emphysematous changes, lung congestion, lung atelectasis and lung haemorrhage were observed in both CTR and MAG groups and were reported to be unlikely to be relevant as they may represent euthanasia/anaesthesia or even post-mortem related changes (Figure S7 A and B).

Materials and methods

Formalin-fixed tissues were provided. The tissued were collected from:

- n=40 mouse pups (heart, lung, liver, spleen, brain)
- n=24 adults/mothers (heart, lung, liver, spleen, brain)
- n=97 fetuses (entire body)
- n=95 corresponding placentas.

The tissue was trimmed and routinely processed for histologic examination (hematoxylin and eosin stain; HE). The histology slides were provided with labels only indicating the basic information about the tissue in order to keep the pathologist blinded. All samples were evaluated by the pathologist in a blinded manner, in order to prevent the reporting of false-positive results.

Results and conclusions

The overall examination of tissue collected from mouse pups and their mothers from the cohort F1 did not reveal any relevant histopathologic changes, with the exception of one pup belonging to the control group that was born with hydrocephalus. Given that only one animal was affected, this lesion is more likely to represent a spontaneous change rather than an experiment-related one.

Liver congestion in three pups belonging to treated group is an unspecific finding and was considered little relevant.

Lung hemorrhage in 5/40 pups was typically mild and may possibly correspond to a euthanasia-related change.

Similar to the F1 cohort, relevant histopathologic changes were not present in the Foetal dev (ED 14.5) cohort. As in F1, mild lung hemorrhage was observed, i.e. in 4 surrogate mothers belonging to treated group as well in 3 surrogate mothers belonging to the control group in the FDev cohort.

Placental tissue revealed mild to moderate necrosis of the decidual layer independently from control or treated group. This finding was interpreted to correspond to a physiologic change as the decidual layer is reported to regress in preparation for birth.

The rare presence of vascular protein casts, potentially compatible with vascular thrombosis, in the labyrinth or giant cell trophoblast layers of three pups was considered little relevant due to the lack of associated tissue damage and the rare occurrence of this change.

Examination of murine fetuses did not reveal any relevant histopathologic changes. The vascular congestion of subcutaneous and liver tissue is a non-specific change and was considered to be of little relevance in this study.

In conclusion, there are no major histopathologic findings in all examined slides from the listed mice and thus no resulting morphologic differences between control and experimental group.

Additional information on mice to F3 generation post static magnetic field embryo transfer (S8)

In order to assess any potential long-term adverse effects of magnetic field (MF) exposure, this study was continued on the second (F2) and third generation (F3). We randomly selected male and female mice from F1 and F2 cohorts originating from Control or Static MF embryos. We let them mate naturally with newly purchased mice to avoid interbreeding. As for F0 surrogate mothers, we monitored the total body weight (TBW) of the F1 (Fig. S8A) and F2 (Fig. S8D) mothers throughout the pregnancy as a general indicator of the animal's well-being, a possible indicator of pregnancy, and, therefore, of natural reproductive potential. In all graphs showing the TBW of the mothers during pregnancy until necropsy, we can appreciate a comparable increase in the TBW of the surrogate mothers after natural mating (NM), suggesting that that both males and females generated from treated embryos could maintain their natural reproductive potential. This observation was further confirmed by the pregnancy rates of F1 (Fig. S8C) and F2 (Fig. S8D) mothers. Both Control and Static MF groups were comparable showing pregnancy rates above 90%. This observation is further corroborated by the fact that no significant difference ($p>0.05$) was detected in the average number of pups born from Control or Static MF in F2 (Fig. S8B) and F3 (Fig. S8E) cohorts. Due to logistics issues, unfortunately, the F2 pups' total body weight could not be assessed precisely at weaning (21 days post-delivery) (Fig. S8H); therefore, it was necessary to subdivide the data according to the actual age of the pups at necropsy (Fig. S8I). After this more accurate analysis, we could not find any significant difference between the two experimental groups as for F1 (Fig. S8G). Furthermore, we weighed the organs fresh immediately at necropsy of the F1 pups (Fig. S8K) and of the F0 surrogate mothers (Fig. S8L). For both F1 pups (Fig. S8K) and the F0 surrogate mothers (Fig. S8L) no statistical significance was observed when comparing the weight of the organs between Control and Stati MF groups ($p>0.05$) except for the weight of the F1 pups brains which results significantly higher in Control mice compared to Static MF ($p=0.0122$). It is important to mention that the dissection of F1 pup brains posed a technical challenge due to the small size of the animals, making it difficult to achieve precise and consistent dissection of such delicate organ. Nonetheless, given that no histopathological defects were detected in either group, the observed weight discrepancy could potentially be attributed to an imprecise dissection technique for this specific organ rather than a direct consequence of Static MF exposure.

Fig. S1.

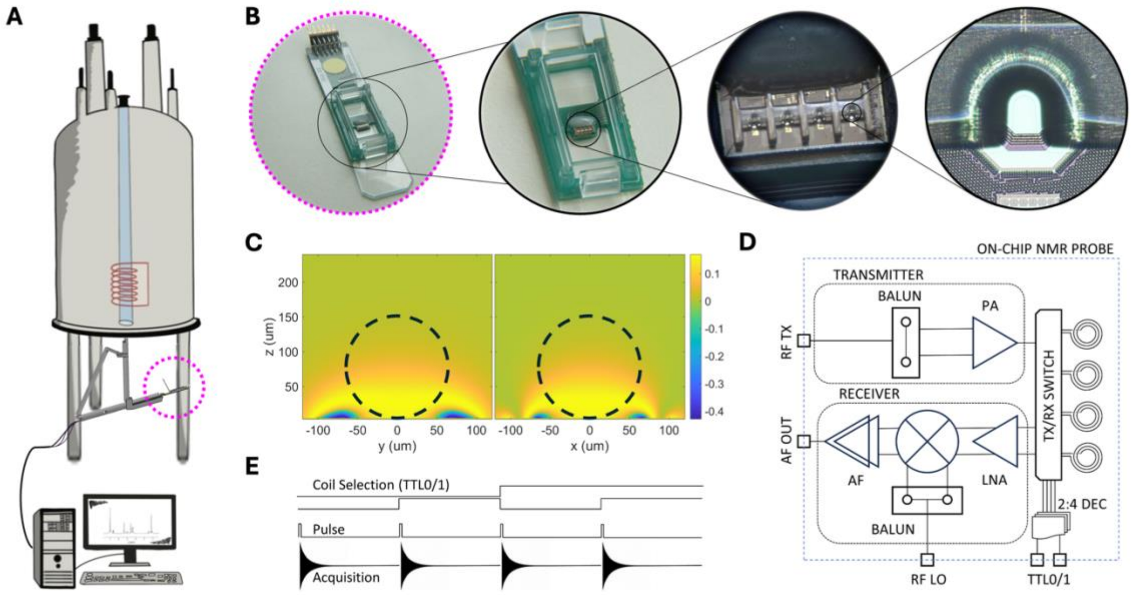

High-throughput magnetic resonance hardware for nanoliter cell cultures analysis (A) Rendering of the Nuclear Magnetic Resonance (NMR) spectrometer comprising a standard 54 mm bore magnet; a custom 3D printed manual loading system for microchip-based NMR sensors; PC and console. (B) Photograph of the microchip-based sensor comprising: a 12-pin connector; a semi-transparent circuit substrate; a chamber to host the culture medium; a 4-channel micro-system designed to host 4 nanoliter-size live samples during measurements. The microchip is glued to the printed circuit board and, on top of it, a 3D-printed micro-structure is glued to align the samples with 4 identical micro-coils. (C) Maps of sensitivity of the integrated microcoil in experimental conditions (τ = 20 μs, I = 2.5 mA) at y = 0 and x = 0 cross-sections. The static B_0 field is oriented along $\overline{x}$, while the $\overline{z}$ axis is perpendicular to the coil surface. The sensing map is computed via the Biot-Savart law considering nutation angles. Dashed shapes indicate the sample positioning. (D) Schematics of the microchip electronics comprising: (1) Differential Transmitter; (2) Heterodyne receiver; (3) 4 identical micro-coils (id = 140 μm, od = 220 μm, 29 loops in total, 6 metal layers); (4) A 2:4 decoder and a radio-frequency switch for coil selection. (E) 1D ^{1}H pulse sequence for pipeline measurements on the 4 channels. Two TTL signals are used for coil selection and sent to the decoder. A TX/RX switch, timed with coil selection, allows for the typical pulse/acquisition scheme.

Fig. S2.

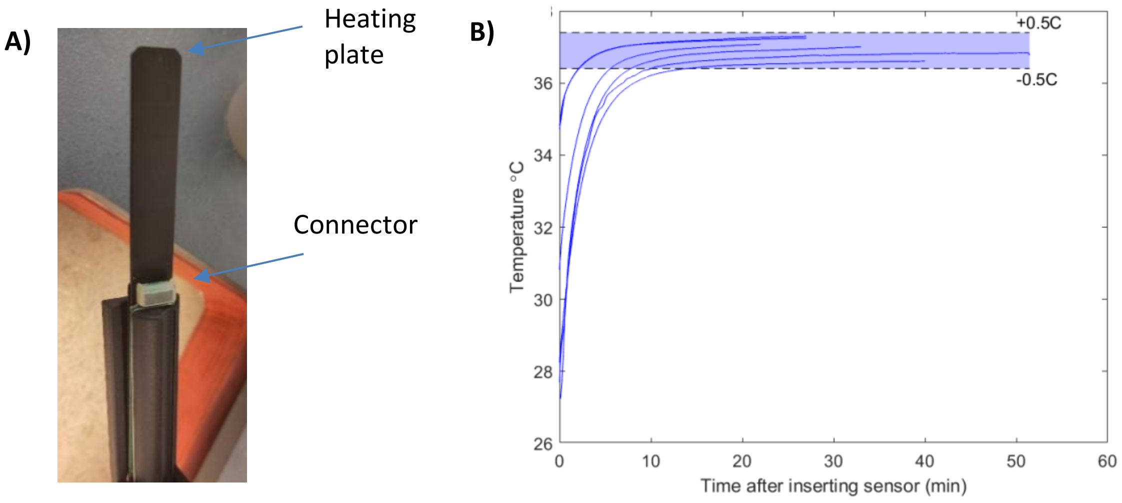

Calibration of temperature variation during measurements: A) Flat heater used to maintain sample temperature. The sensor is held against the heater by the electrical connector at the bottom and a clip at the top. B) Temperature calibration read-out from thermocouple placed in proximity of the sensing region on repeated insertion of the sensor into the magnet.

Fig. S3.

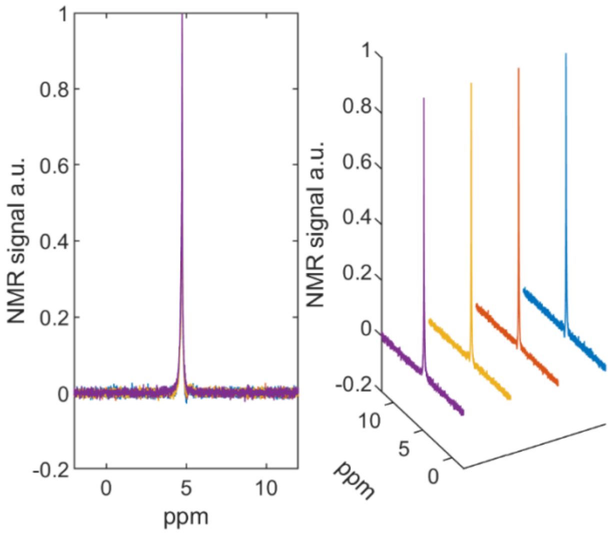

Shimming of the 4-channel MRS setup. Overlapped (left) and distributed (right) water peaks.

Fig. S4.

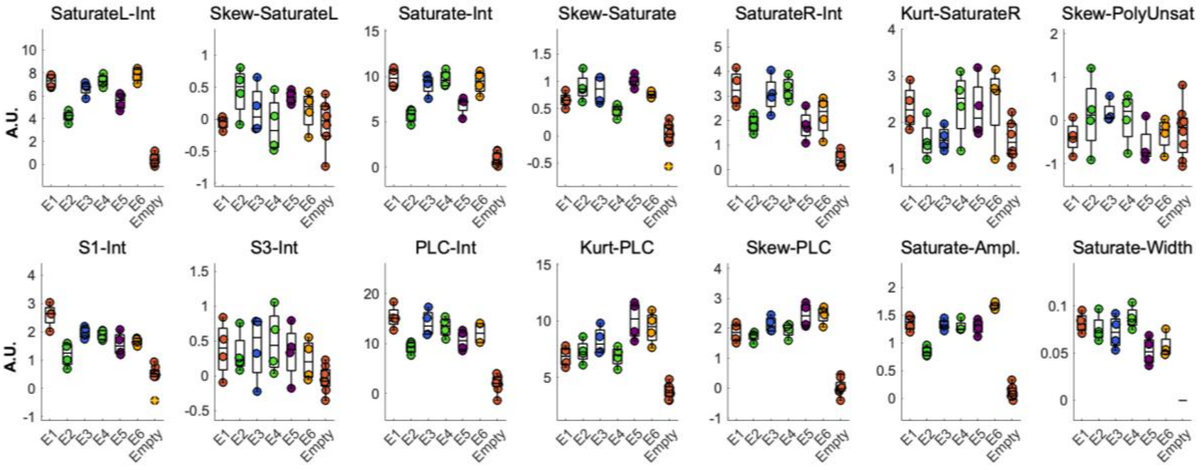

Repeated measurements on single 8-cell bovine embryos. Biomarkers quantification for 6 embryos ("E1" to "E6") and culture medium ("Empty"). Biomarkers include Intensity (-Int), Skewness (-Skew), and Kurtosis (-Kurt) within specific chemical shift regions of the spectrum: PLC [0.83-2.8], Saturate [1.1-1.5], S1 [0.83-1.03], S3 [2.23-2.36], SaturateL [1.3-1.5], SaturateR [1.1-1.3]. Saturate-Ampl and Saturate-Width are derived from Lorentzian fit parameters in the Saturate region.

Fig. S5.

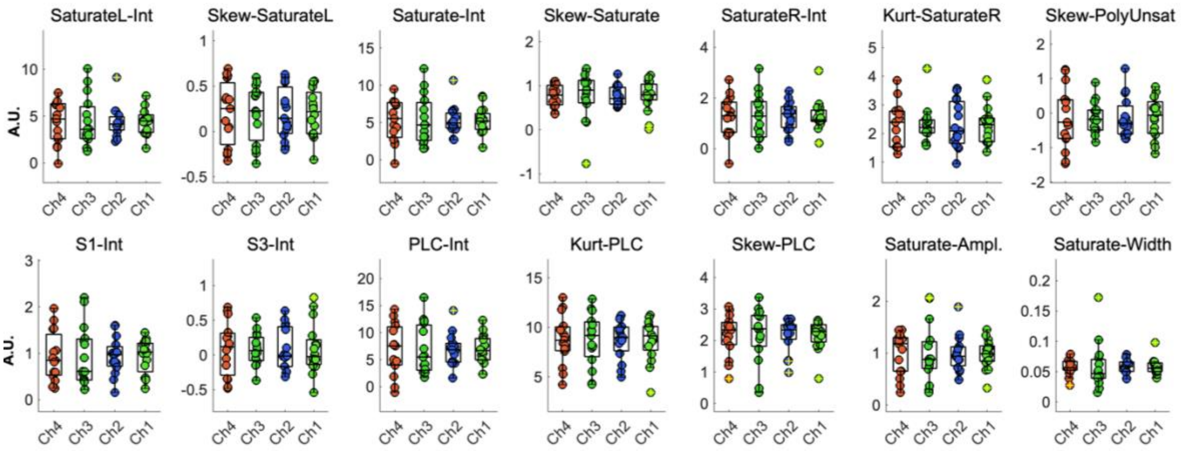

MRS biomarkers of 8-cell embryos VS sensor channel ID. Biomarkers quantification for the 61 embryos represented in Fig. 2, plotted by channel ID “1” (n=16), “2” (n=15), “3” (n=15), “4” (n=15). Biomarkers include Intensity (-Int), Skewness (-Skew), and Kurtosis (-Kurt) within specific chemical shift regions of the spectrum: PLC [0.83-2.8], Saturate [1.1-1.5], S1 [0.83-1.03], S3 [2.23-2.36], SaturateL [1.3-1.5], SaturateR [1.1-1.3]. Saturate-Ampl and Saturate-Width are derived from Lorentzian fit parameters in the Saturate region.

Fig. S6.

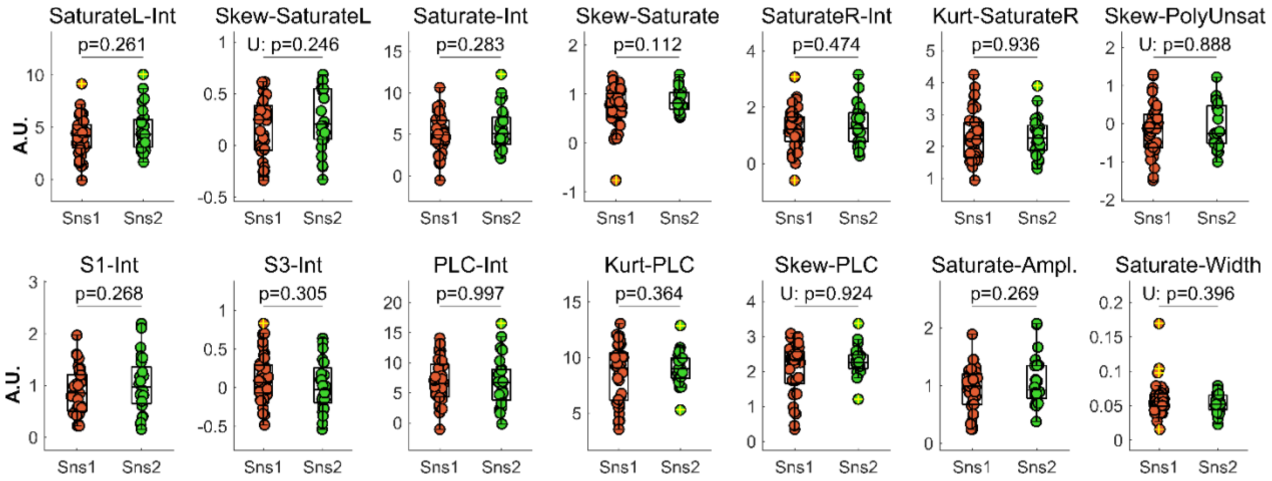

MRS biomarkers of 8-cell embryos VS sensor ID. Biomarkers quantification for the 61 embryos represented in Fig. 2, plotted by sensor ID “Sns 1” (n=37) and “Sns 2” (n=24). Normality assessed with Lilliefors test, with significance determined by t-test or Mann-Whitney U Test (U). Biomarkers include Intensity (-Int), Skewness (-Skew), and Kurtosis (-Kurt) within specific chemical shift regions of the spectrum: PLC [0.83-2.8], Saturate [1.1-1.5], S1 [0.83-1.03], S3 [2.23-2.36], SaturateL [1.3-1.5], SaturateR [1.1-1.3]. Saturate-Ampl and Saturate-Width are derived from Lorentzian fit parameters in the Saturate region.

Fig. S7.

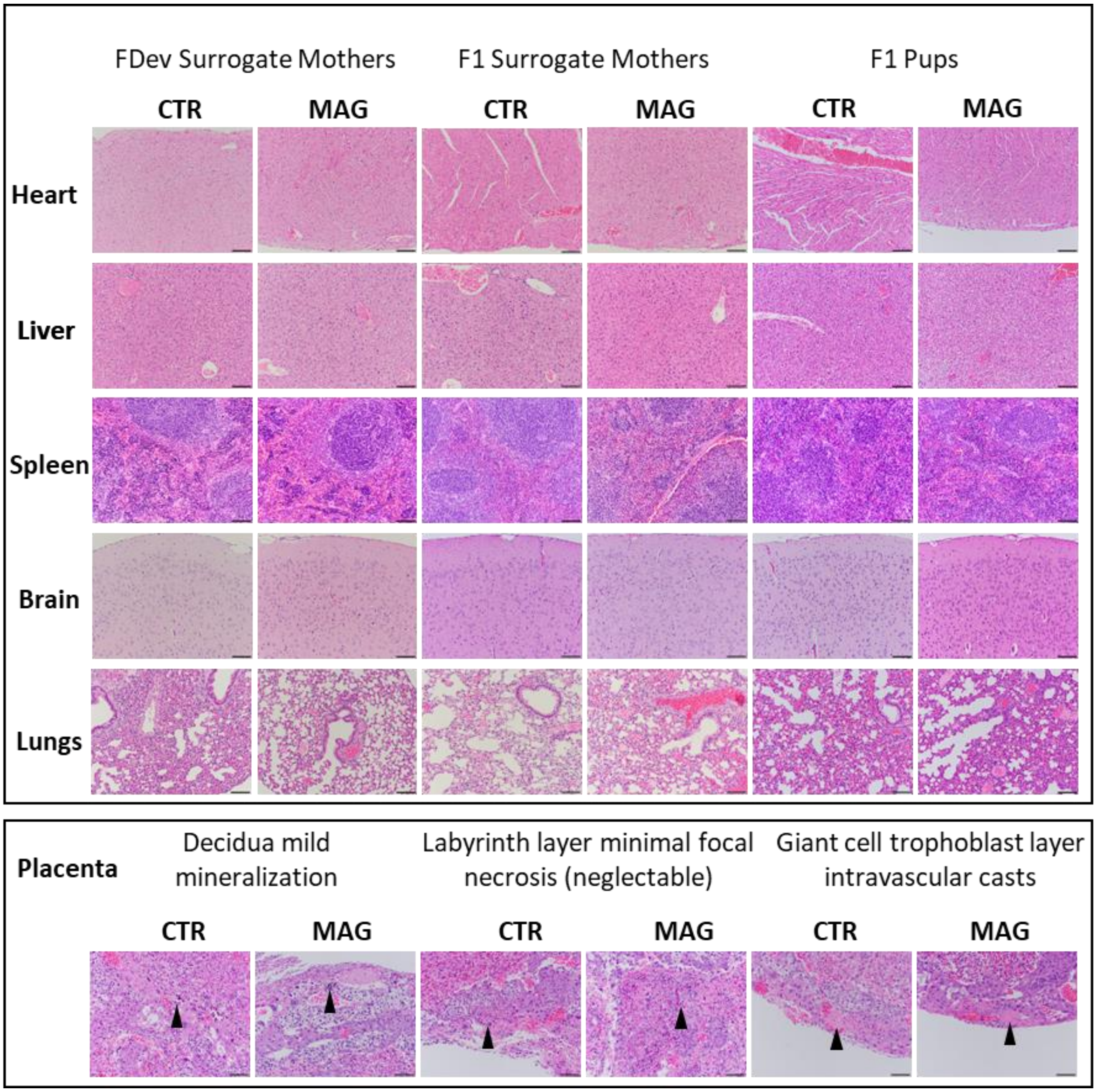

S7-A: Bright field micrographs of H&E stained tissue sections of the five selected organs and the placenta for post-mortem analysis. Magnification 20X. Scale bar 100um. Arrows indicate lesions.

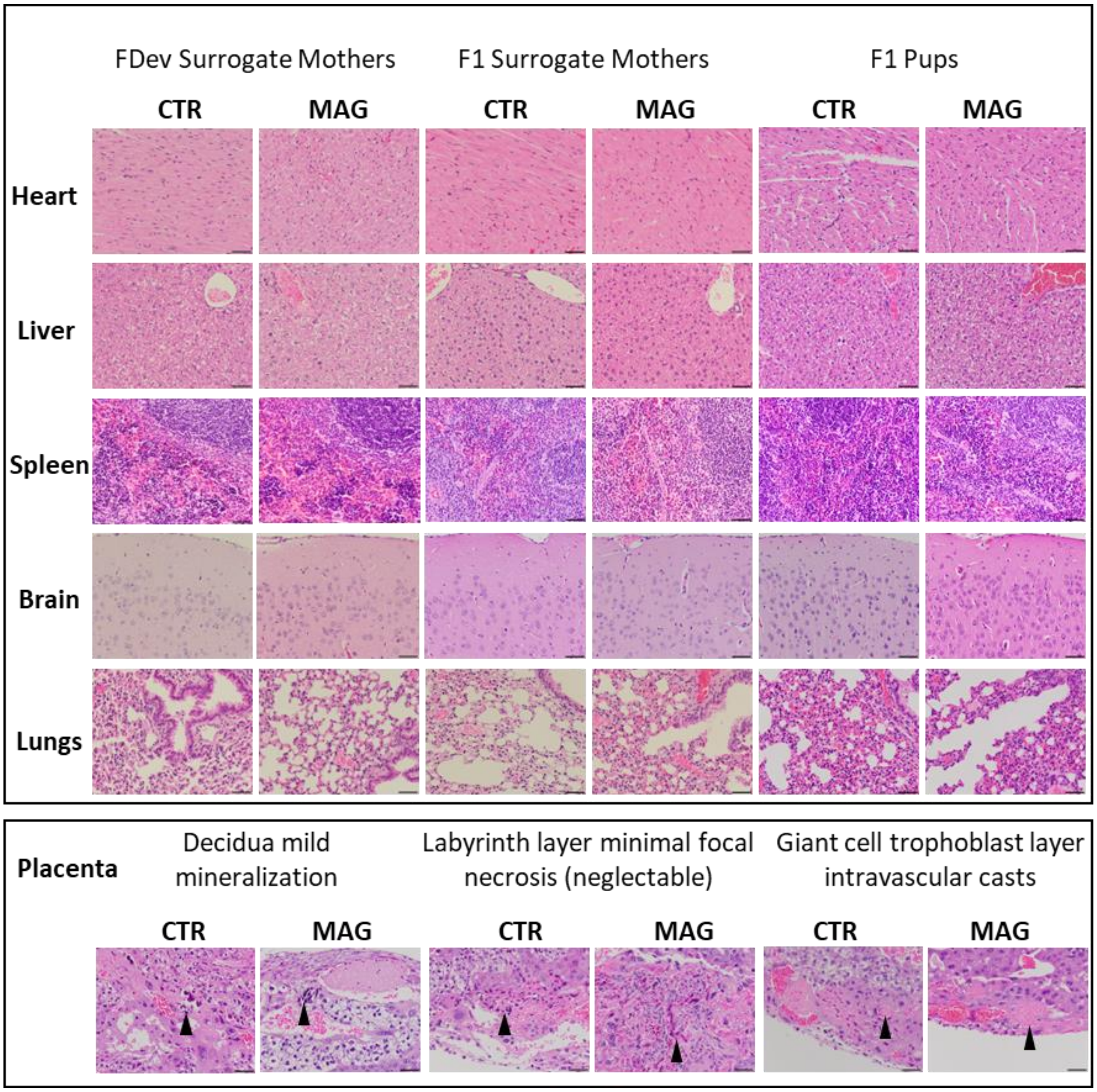

S7-B: Bright field micrographs of H&E stained tissue sections of the five selected organs and the placenta for post-mortem analysis. Magnification 40X. Scale bar 100um. Arrows indicate lesions.

Fig. S8.

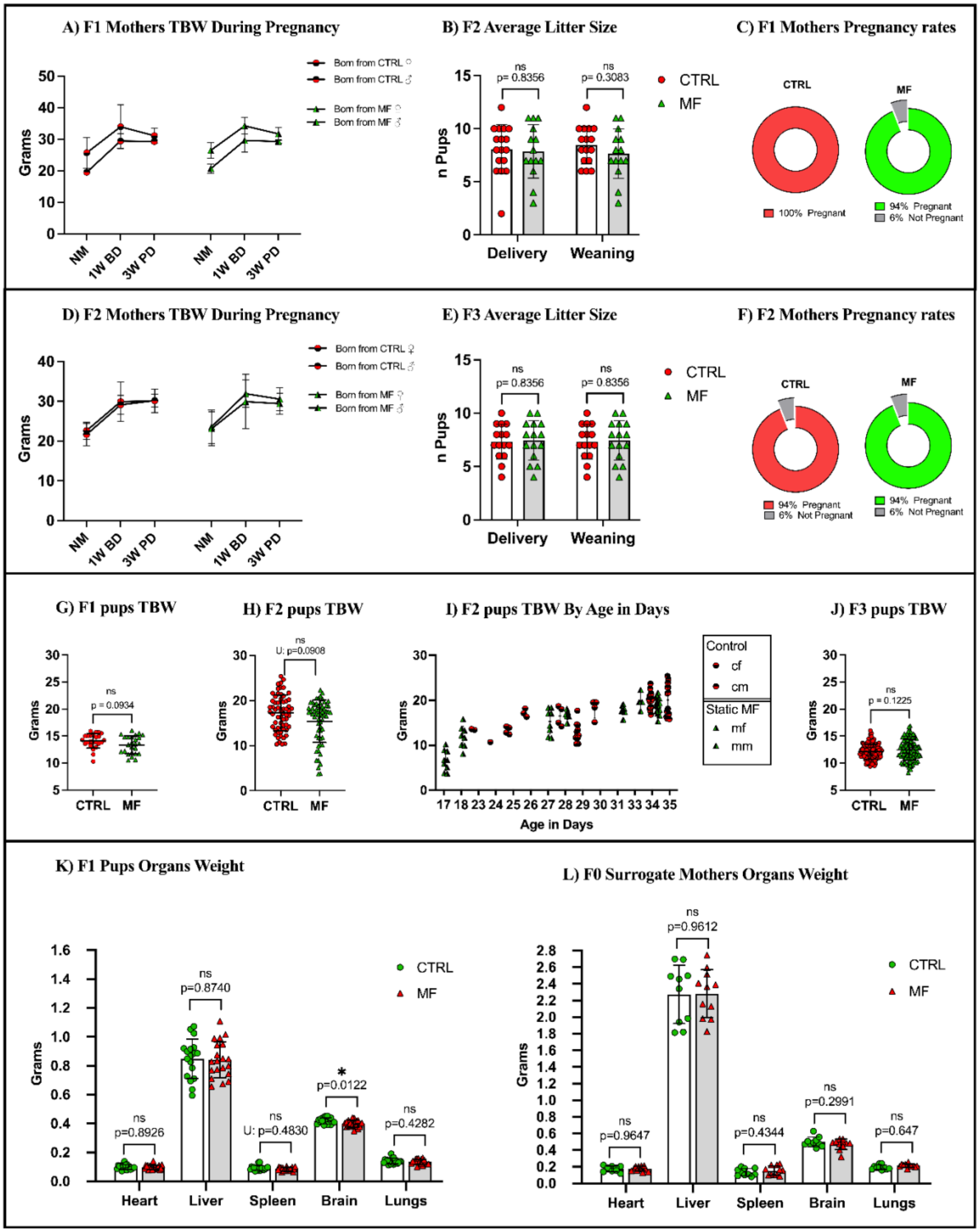

Static Magnetic Field (MF) exposure of 2-cell embryos does not impair the development of mice up to F3. For all the comparisons statistical significance was set at $p<0.05$. Bars represent SD. p

values are indicated on top of each comparison. Normality assessed with Kolmogorov-Smirnov, with significance determined Unpaired T-test with Welch's correction or Mann-Whitney U Test (U). (A) Total body weight of F1 mothers of F2 cohort during pregnancy up to weaning after natural mating (CF = control females n = 11; CM = control males n = 6, MF = Static MF females n = 8; MM = Static MF Males n = 6; NM = Natural Mating, 1WBD = 1 week before delivery, 3WPD = 3 weeks post delivery). (B) Average litter size of F2 pups delivered from surrogate mothers post natural mating at delivery and weaning (3 weeks post delivery). (At Delivery: n= 17 Control F1 Mothers, n = 137 Control F2 Pups; n = 14 Static MF Mothers; n = 110 Static MF Pups. At Weaning: n= 17 Control F1 Mothers, n = 135 Control F2 Pups; n = 14 Static MF Mothers; n = 107 Static MF Pups). (C) Pregnancy rates of F1 mothers expressed as percentage of pregnant mothers after natural mating (NM) for the Control group (n = 12 Control Mothers) and the Static Magnetic Field group (n= 17 MF Mothers). (D) Total body weight of F2 mothers of F3 cohort during pregnancy up to weaning after natural mating (CF = control females n = 7; CM = control males n = 8, MF = Static MF females n = 7; MM = Static MF Males n = 8; NM = Natural Mating, 1WBD = 1 week before delivery, 3WPD = 3 weeks post delivery). (E) Average litter size of F3 pups delivered from surrogate mothers post natural mating at delivery and weaning (3 weeks post delivery. (n= 15 Control F2 Mothers, n = 110 Control F3 Pups; n = 14 Static MF Mothers; n = 112 Static MF Pups). (F) Pregnancy rates of F2 mothers expressed as percentage of pregnant mothers after natural mating (NM) for the Control group (n = 16 Control Mothers) and for the Static Magnetic Field group (n = 15 MF Mothers). (G) F1 pups total body weight at weaning (3 weeks post delivery). (n= 24 Control, n = 25 Static MF). (H) F2 pups total body weight at weaning (3 weeks post delivery). (n= 60 Control, n = 62 Static MF). (I) Total body weight of Control and Static MF F2 pups according to age in days at necropsy and subgroups (CF = control females; CM = control males, MF = Static MF females; MM = Static MF Males) to explain the large spread and increase in total body weight over time observed in figure H). (J) F3 pups total body weight at weaning (3 weeks post delivery). (n= 110 Control, n = 112 Static MF).(K) F1 Pups organs weight at weaning. (n= 18 Control, n = 20 Static MF). (L) F0 Surrogate Mothers organs weight at necropsy (3 weeks post delivery). (n= 10 Control, n = 11 Static MF).

Fig S9.

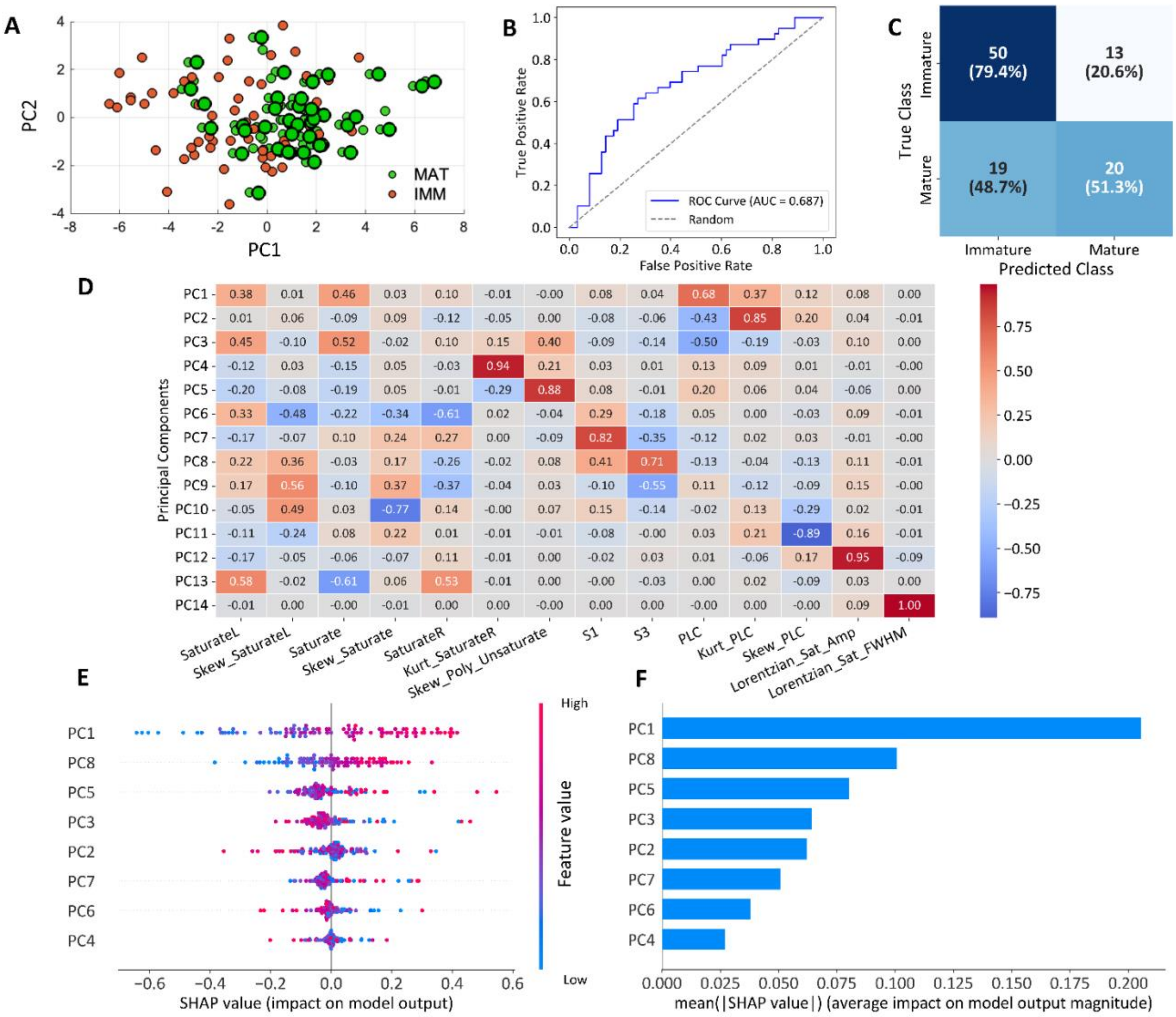

Classification of oocytes maturation based on MRS biomarkers. **(A)** Typical first and second principal components [A.U] observed after applying SMOTE (Synthetic Minority Over-sampling Technique) to balance the dataset (N_{TOT}=102, N_{IMM}=63, N_{MAT}=39). Red: immature oocytes; big green: original mature oocytes; small green: synthetic mature oocytes. **(B)** ROC curve with AUC comparison between a dummy classifier (predicting the most frequent class) and our SVM model, both evaluated using a CVLOO strategy. **(C)** Confusion matrix aggregated from the CVLOO predictions of the SVM model. SMOTE was applied exclusively to the training data in each fold to address class imbalance, ensuring no data leakage into the validation set. **(D)** PCA loadings showing the contribution of each original feature to the principal components. PCA was performed on the training data prior to SMOTE application. **(E-F)** SHAP summary and bar plots illustrating the first 8 most influential principal components in the model and their effect on prediction outcomes.

Fig S10.

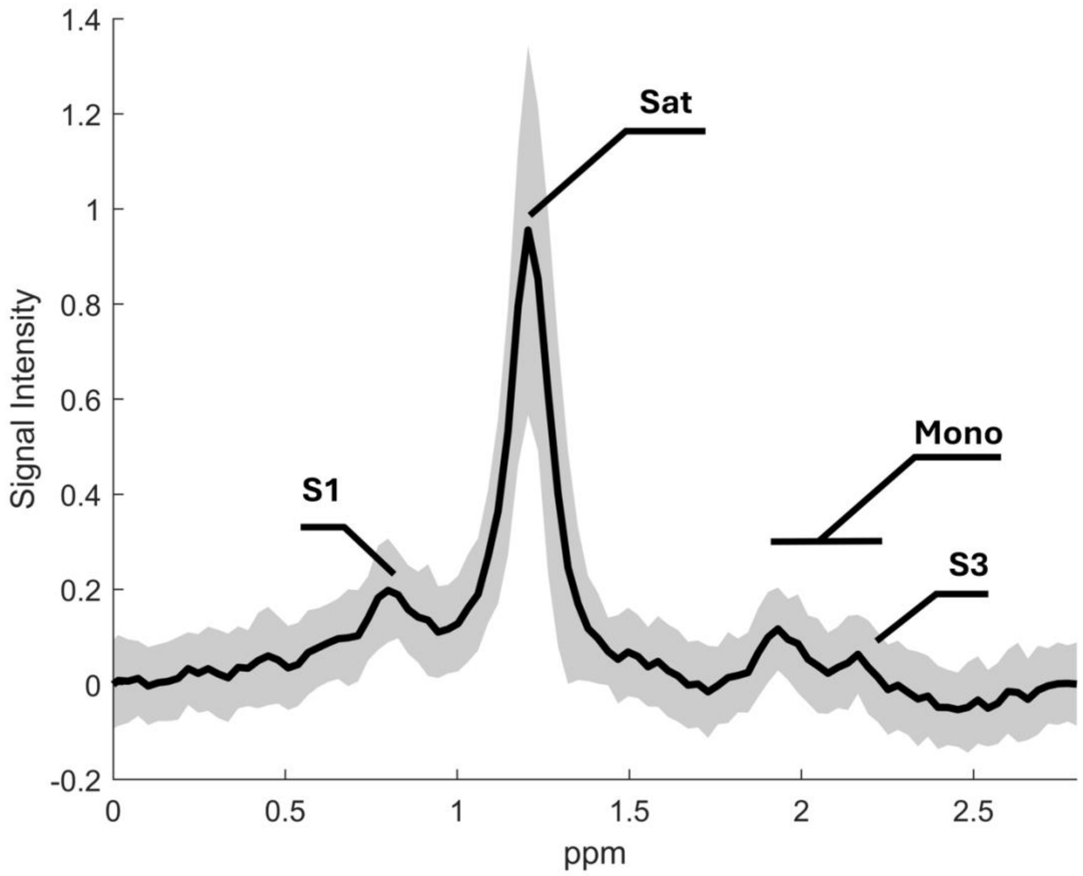

Spectral signal distribution between 0 and 2.8 ppm. The global mean spectrum across all samples is displayed, with the standard deviation (shaded area) shown to indicate variability. This representation highlights characteristic spectral features associated with specific biomarkers. Key biomarkers regions corresponding to saturated lipids (Sat), monounsaturated lipids (Mono), and specific signals (S1, S3) are indicated.

Fig S11.

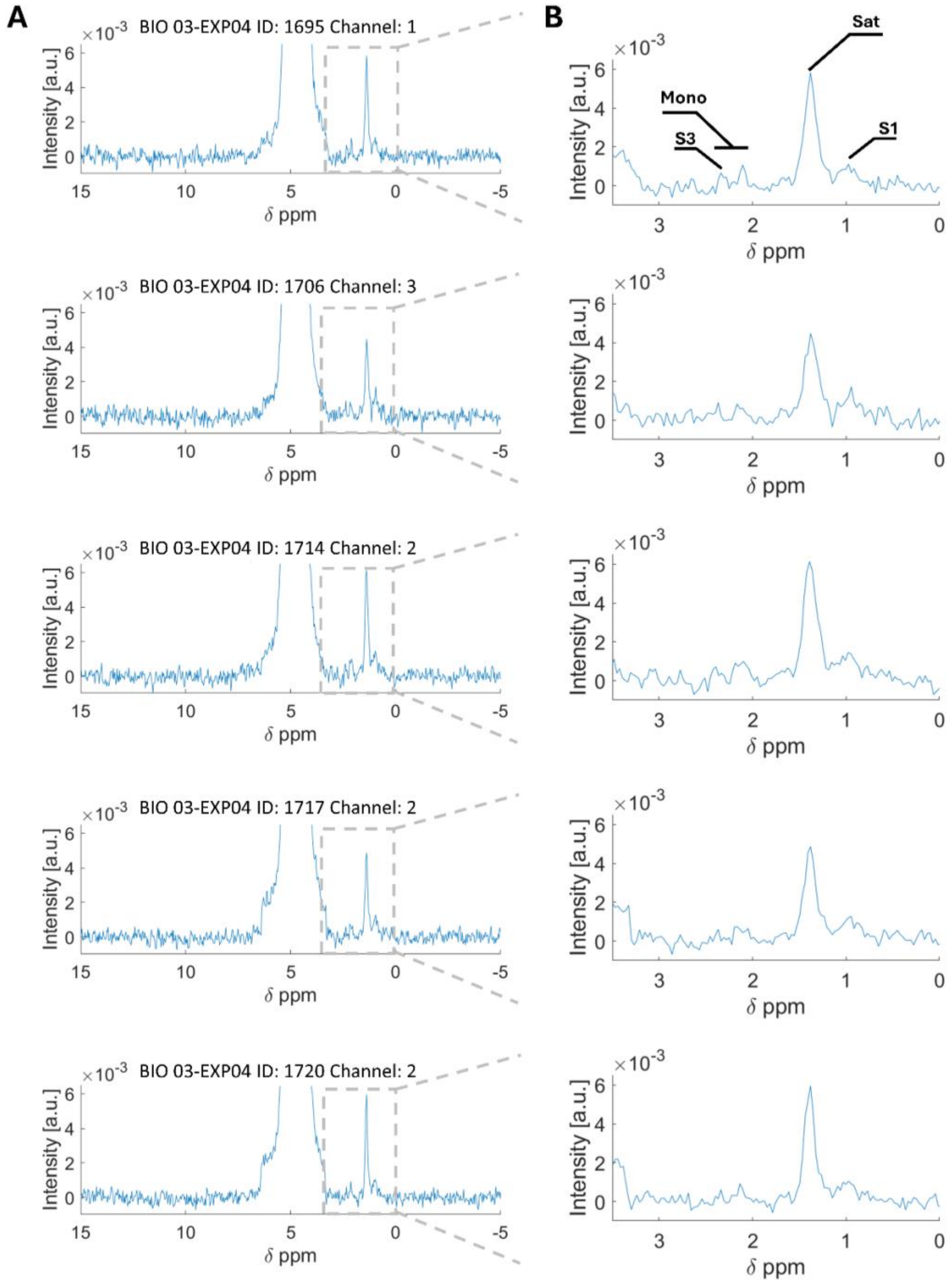

Spectral signal representation across selected samples. Key biomarkers regions are indicated. **(A)** Representative ^{1}H NMR spectra from five samples displaying high-intensity saturated lipid peaks. Full spectra are shown over the -5 to 15 ppm range. **(B)** Enlarged view of the 0 to 3.5 ppm region, emphasizing spectral regions relevant to biomarker identification. All pre-processed plots available at the following LINK.

Fig S12.

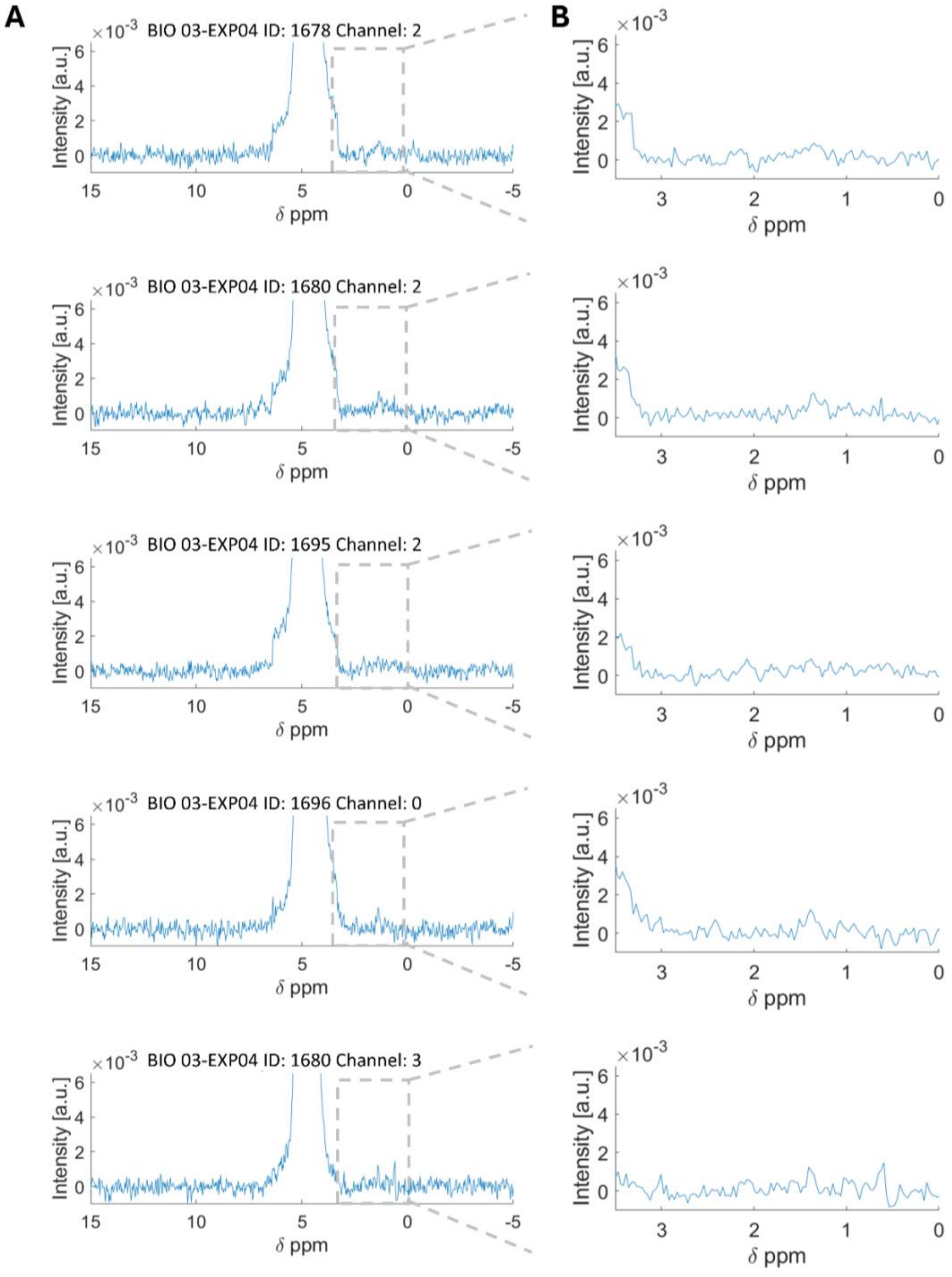

Spectral signal representation across selected samples. **(A)** Representative ^{1}H NMR spectra from five samples displaying barely any saturated lipid peaks. Full spectra are shown over the -5 to 15 ppm range. **(B)** Enlarged view of the 0 to 3.5 ppm region, emphasizing spectral region relevant to biomarker identification. All pre-processed plots available at the following LINK.

Table S1.

Chemical group	^{1}H Shift (δ ppm)
$CH_3 -$	0.83–1.03
$-(CH_2)_n$	1.22–1.42
$- CH_2 - CH_2 - CO_2 -$	1.52–1.70
$- CH = CH - CH_2 -$	1.94–2.14
$- CH_2 - CO_2 -$	2.23–2.36
$= CH - CH_2 - CH =$	2.70–2.84

Lipids chemical shifts as reported in literature. REF: Knothe, G., & Kenar, J. A. (2004). Determination of the fatty acid profile by 1H-NMR spectroscopy. *European Journal of Lipid Science and Technology*, *106*(2), 88-96.

Table S2.

Biomarker Name	Range (ppm)	Type	Definition
SaturateL	[1.3 – 1.5]	Integral	Integral intensity of the left portion of the saturated lipid peak
Skew_SaturateL	[1.3 – 1.5]	Skewness	Skewness of the left portion of the saturated lipid peak
Saturate	[1.1 – 1.5]	Integral	Integral intensity of the entire saturated lipid peak
Skew_Saturate	[1.1 – 1.5]	Skewness	Skewness of the entire saturated lipid peak
SaturateR	[1.1 – 1.3]	Integral	Integral intensity of the right portion of the saturated lipid peak
Kurt_SaturateR	[1.1 – 1.3]	Kurtosis	Kurtosis of the right portion of the saturated lipid peak
Skew_Poly_Unsaturate	[2.6 – 2.8]	Skewness	Skewness of the polyunsaturated lipid peak
S1	[0.83 – 1.03]	Integral	Integral intensity of spectral region of interest named S1
S3	[2.33 – 2.36]	Integral	Integral intensity of spectral region of interest named S3
PLC	[0.83 – 2.8]	Integral	Integral intensity of the proportional lipid content (PLC)
Kurt_PLC	[0.83 – 2.8]	Kurtosis	Kurtosis of the proportional lipid content (PLC)
Skew_PLC	[0.83 – 2.8]	Skewness	Skewness of the proportional lipid content (PLC)
Lorentzian_Sat_Amp	[1.1 – 1.5]	Lorentzian fit parameter	Amplitude of the saturated lipid peak from Lorentzian curve fitting
Lorentzian_Sat_FWHM	[1.1 – 1.5]	Lorentzian fit parameter	Full Width at Half Maximum (FWHM) of the saturated lipid peak from Lorentzian curve fitting

List of Biomarkers acronym and their definition.

Table S3

Experimental Group	Developmental Category	N	Percentage	Grouped Percentage
MRS ($N_{TOT} = 64$)	E	21	33%	80%
	L	30	47%	
	AB	5	8%	20%
	B	8	12%	
CONTROL ($N_{TOT} = 20$)	E	3	15%	75%
	L	12	60%	
	AB	1	5%	25%
	B	4	20%	

Developmental Category	Representative Pictures			
E	Early Arrested Embryo	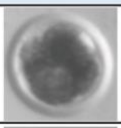		
L	Late Arrested Embryo	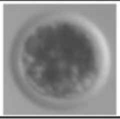		
AB	Early Arrested Blastocyst	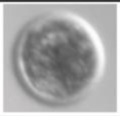		
B	Expanded Blastocyst	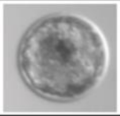	Fully Hatched Blastocyst	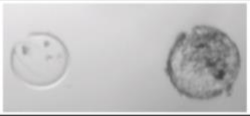

Rounded percentage of embryos that reached developmental milestones in CTRL and MRS groups.

Movie S1.

Sensor preparation protocol.
